# Lipid-Based Nutrient Supplementation Increases High-Density Lipoprotein (HDL) Cholesterol Efflux Capacity and Is Associated with Changes in the HDL Glycoproteome in Children

**DOI:** 10.1021/acsomega.1c04811

**Published:** 2021-11-18

**Authors:** Brian V. Hong, Chenghao Zhu, Maurice Wong, Romina Sacchi, Christopher H. Rhodes, Jea Woo Kang, Charles D. Arnold, Seth Adu-Afarwuah, Anna Lartey, Brietta M. Oaks, Carlito B. Lebrilla, Kathryn G. Dewey, Angela M. Zivkovic

**Affiliations:** † Department of Nutrition, University of California, Davis, Davis 95616, California, United States; ‡ Department of Chemistry, University of California, Davis, Davis 95616, California, United States; § Department of Nutrition and Food Science, University of Ghana, Legon LG 134, Ghana; ∥ Department of Nutrition and Food Sciences, University of Rhode Island, Kingston 02881-2003, Rhode Island, United States

## Abstract

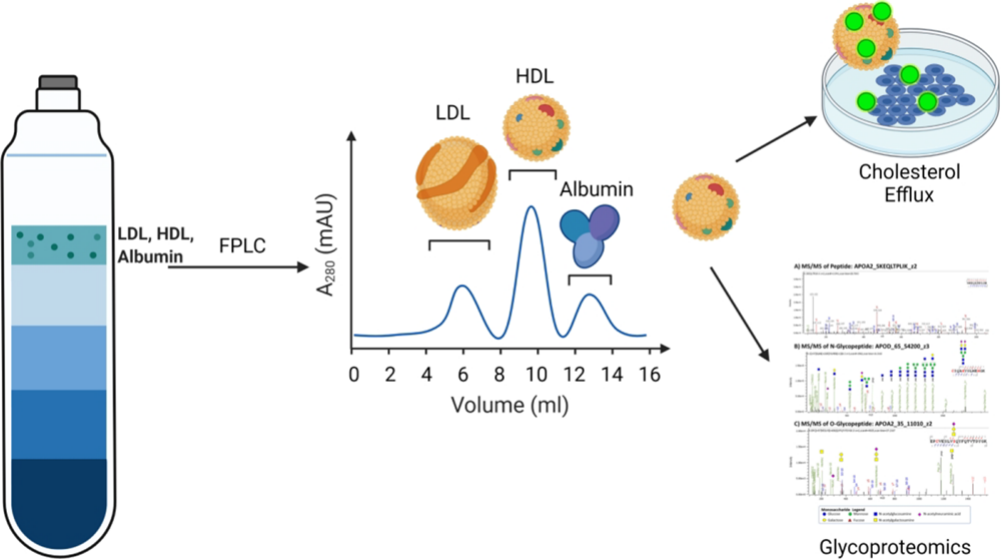

Prenatal plus postnatal small-quantity lipid-based nutrient supplements (SQ-LNS) improved child growth at 18 months in the International Lipid-Based Nutrient Supplements DYAD trial in Ghana. In this secondary outcome analysis, we determined whether SQ-LNS versus prenatal iron and folic acid (IFA) supplementation improves the cholesterol efflux capacity (CEC) of high-density lipoprotein (HDL) particles and alters their lipidomic, proteomic, or glycoproteomic composition in a subset of 80 children at 18 months of age. HDL CEC was higher among children in the SQ-LNS versus IFA group (20.9 ± 4.1 vs 19.4 ± 3.3%; one-tailed *p* = 0.038). There were no differences in HDL lipidomic or proteomic composition between groups. Twelve glycopeptides out of the 163 analyzed were significantly altered by SQ-LNS, but none of the group differences remained significant after correction for multiple testing. Exploratory analysis showed that 6 out of the 33 HDL-associated proteins monitored differed in glycopeptide enrichment between intervention groups, and 6 out of the 163 glycopeptides were correlated with CEC. We conclude that prenatal plus postnatal SQ-LNS may modify HDL protein glycoprofiles and improve the CEC of HDL particles in children, which may have implications for subsequent child health outcomes. This trial was registered at clinicaltrials.gov as NCT00970866.

## Introduction

Inadequate micronutrient intake is common in low- to middle-income countries and is associated with adverse consequences including slower linear growth of children,^1^ impaired cognitive development, and diseases in later stages of life.^2^ In addition, n-3 and n-6 polyunsaturated fatty acids may be low in food sources and breast milk in certain countries.^3^ The International Lipid-Based Nutrient Supplements (iLiNS) project developed small-quantity lipid-based nutrient supplements (SQ-LNS) to enrich home-prepared foods, particularly for women and children.^4^ SQ-LNS provide vitamins, minerals, and essential fatty acids (linoleic acid and α-linolenic acid (ALA)) to either infants or mothers during pregnancy and lactation.^4^ In Ghana, prenatal SQ-LNS led to increased weight, length, and head circumference among infants of primiparous mothers, compared to those whose mothers received an iron and folic acid (IFA) or multiple micronutrient (MMN) supplement,^5^ and improved the length, weight, and stunting prevalence in the entire cohort at 18 months of age.^6^

High-density lipoprotein (HDL) particles are lipid–protein complexes in circulation that play essential roles in maintaining lipid and cholesterol metabolic homeostasis.^7^ In addition to the well-known atheroprotective effects of HDL,^7,8^ including the role of HDL particles in the regulation of cellular cholesterol concentrations via cholesterol efflux,^9^ recent evidence suggests that HDL particles may also be important for the mother–child dyad. Very little is known about the role of HDL during pregnancy and in early development. In the United States, low maternal HDL cholesterol (HDL-C) was associated with a low birth weight z-score,^10^ and in Ghana, we previously reported that high HDL-C at 36 weeks gestation was positively associated with the duration of gestation.^11^ Even less is known about HDL particles, particularly in infants; however, limited evidence in preterm infants indicates that a lower concentration of HDL particles is found in infants with chronic lung disease compared to those without lung disease.^12^ HDL particles display a variety of immunomodulatory capabilities,^13^ including boosting the ability of innate immune cells to fight infection,^14^ and may thus be particularly important in settings with high infection burden.

The functionality of HDL particles is known to be dictated by their composition, including both the lipid and protein components,^15−17^ and these components are modifiable by diet.^18,19^ We have previously demonstrated that in addition to the proteins and lipids, the glycan components may also play an important role in determining the functional capacity of HDL particles.^20,21^ Specific components such as ALA, an essential n-3 polyunsaturated fatty acid, have been shown to improve the cholesterol efflux capacity (CEC) of HDL particles in vitro.^22^ We have also demonstrated that the glycoprofiles of specific HDL-associated proteins are associated with HDL CEC and can be modified by diet.^21^

Because SQ-LNS deliver essential fatty acids along with proteins and other fats, they may improve the composition and function of HDL particles. In this pilot study and secondary outcome analysis of samples from the iLiNS-DYAD-Ghana trial, we hypothesized that SQ-LNS provided to the mother during pregnancy and the first 6 months postpartum, followed by SQ-LNS provided to the infant from 6 to 18 months of age, would increase HDL CEC by altering HDL lipidomic and glycoproteomic composition.

## Results

The baseline characteristics of the mothers of the 80 selected children, and the child morbidity variables from 6 to 18 months, are presented in Table 1, by intervention group. There were no significant differences in the baseline characteristics between the SQ-LNS and IFA groups. Child growth status at 18 months and the change in z-scores from 12 to 18 months are presented in Table 2, by intervention group. The children in the SQ-LNS group had an increase instead of a decrease in LAZ from 12 to 18 months, and this difference in change of LAZ score was statistically significant (*p* = 0.044).

**Table 1:** Background Characteristics at Enrollment of Mothers in the IFA and SQ-LNS Groups and Child Morbidity from 6 to 18 months in This Subsample^a^

	IFA (*n* = 40)	SQ-LNS (*n* = 40)	*p*-value
background characteristics^a^			
age, year	22.1 ± 3.1 (40)	23.3 ± 3.6 (40)	0.109
estimated prepregnancy BMI^b^, kg/m^2^	21.7 ± 2.0 (40)	21.4 ± 2.0 (36)	0.564
years of formal education, year	8.3 ± 2.7 (40)	8.3 ± 3.1 (40)	1.000
mother’s height, cm	158.1 ± 5.0 (40)	160.4 ± 5.9 (36)	0.065
household food insecurity access score	1.7 ± 3.5 (39)	0.9 ± 2.6 (40)	0.241
gestational age at enrollment, week	39.0 ± 2.2 (40)	39.5 ± 1.7 (40)	0.228
asset index^c^	0.02 ± 0.86 (40)	0.01 ± 0.89 (40)	0.952
housing index^c^	–0.27 ± 1.07 (40)	0.05 ± 0.87 (40)	0.139
maternal malaria RDT^d^	4/40 (10.0)	6/40 (10.0)	0.505
mother’s blood hemoglobin conc., g/L	109.1 ± 11.0 (40)	107.6 ± 9.8 (40)	0.521
child morbidity variables from 6 to 18 months			
any illness episodes	12.4 ± 5.2 (36)	13.9 ± 7.5 (39)	0.327
fever episodes	2.0 ± 1.7 (36)	2.3 ± 2.2 (39)	0.529
loose stool episodes	2.2 ± 2.4 (36)	3.2 ± 3.2 (39)	0.114
respiratory infection episodes	7.8 ± 3.4 (36)	7.7 ± 3.8 (39)	0.945
poor appetite episodes	3.1 ± 2.5 (36)	3.6 ± 3.3 (39)	0.465

a Values are presented as mean ± SD (*n*). Values are presented as *n*/*N* = number of participants whose response was “yes” in question/*n* of participants analyzed. RDT, rapid diagnostic test; IFA, iron and folic acid; SQ-LNS, small-quantity lipid-based nutrient supplement.

b Prepregnancy body mass index (BMI) was estimated from height and weight at enrollment using polynomial regression with gestational age, gestational age squared, and gestational age cubed as predictors. Mean-estimated prepregnancy BMI in this subcohort was lower than in the larger study population^6^ because of the selection criteria for this subcohort (nonoverweight women).

c Proxy indicators for household socioeconomic status. A higher index value means higher socioeconomic status.

d Clearview Malarial Combo, Vision Biotech.

**Table 2:** Anthropometric Characteristics of Children in the Subsample, by the Intervention Group^a^

	Z-score at 18 months			change in the z-score from 12 to 18 months		
growth outcomes	IFA (*n* = 40)	SQ-LNS (*n* = 40)	*p*-value	IFA (*n* = 40)	SQ-LNS (*n* = 39)	*p*-value
WAZ	–0.94 ± 1.01 (40)	–0.69 ± 1.09 (40)	0.304	–0.14 ± 0.41 (37)	–0.05 ± 0.54 (35)	0.430
LAZ	–0.97 ± 0.91 (40)	–0.63 ± 1.11 (40)	0.138	–0.16 ± 0.36 (37)	0.05 ± 0.47 (35)	**0.044**
HCZ	–1.16 ± 1.04 (40)	–1.08 ± 0.87 (39)	0.717	–0.30 ± 0.50 (37)	–0.24 ± 0.44 (34)	0.647
WLZ	–0.66 ± 1.06 (40)	–0.54 ± 1.05 (40)	0.635	–0.10 ± 0.57 (37)	–0.11 ± 0.59 (35)	0.944

a Values are represented as mean ± SDs (*n*). IFA, iron and folic acid; SQ-LNS, small-quantity lipid-based nutrient supplement; WAZ, weight for age z-score; LAZ, length for age z-score; HCZ, head circumference for age z-score.

### HDL Composition and Function by the Intervention Group

The primary HDL outcome variables are shown in Table 3. HDL CEC was significantly higher among children in the SQ-LNS group compared to those in the IFA group (*p* = 0.038, one-tailed test). HDL lipidome characteristics including EOD_18_, average chain length (ACL), and surface/core lipid ratio were not significantly different between groups (Table 3). HDL APOA1, SAA1, SAA2, and APOL1 were also not significantly different by intervention group.

**Table 3:** Primary HDL Outcomes at 18 months of Age, by the Intervention Group^a^

			unadjusted model		adjusted model^e^	
variable	IFA (*N* = 40)	SQ-LNS (*N* = 40)	difference in means (95% CI)	*p*-value	difference in means (95% CI)	*p*-value
cholesterol efflux (%)^f^	19.4 ± 3.3	20.9 ± 4.1	1.5(−0.2,3.2)	**0.038**	1.5(−0.2,3.2)	**0.038**
overall EOD_18_^b^^,^^g^	1.4 ± 0.1	1.4 ± 0.1	0.0(−0.0,0.1)	0.429	0.0(−0.0,0.1)	0.429
overall ACL^b^^,^^g^	15.6 ± 0.4	15.6 ± 0.5	–0.0(−0.2,0.2)	0.960	–0.0(−0.2,0.2)	0.831
surface/core lipid ratio^c^^,^^g^	2.0 ± 0.3	2.0 ± 0.4	0.0(−0.2,0.2)	0.942	0.0(−0.2,0.2)	0.942
APOA1^d^^,^^g^	1.4 ± 0.5 × 10^6^	1.5 ± 0.5 × 10^6^	0.1( – 0.1,0.3) × 10^6^	0.410	0.1( – 0.1,0.3) × 10^6^	0.410
SAA1^g^	8.4 ± 5.9 × 10^3^	9.5 ± 5.7 × 10^3^	1.1( – 1.4,3.7) × 10^3^	0.387	1.9( – 0.7,4.5) × 10^3^	0.146
SAA2^g^	1.4 ± 0.9 × 10^4^	1.6 ± 1.0 × 10^4^	0.2( – 0.2,0.6) × 10^4^	0.344	0.2( – 0.2,0.6) × 10^4^	0.344
APOL1^g^	8.6 ± 8.6 × 10^3^	7.8 ± 6.5 × 10^3^	–0.7( – 4.1,2.7) × 10^3^	0.671	0.3( – 3.0,3.6) × 10^3^	0.864

a Values are represented as mean ± SDs (*n*). IFA, iron and folic acid; SQ-LNS, small-quantity lipid-based nutrient supplement.

b EOD_18_: Equivalent of double-bond per 18 carbons; ACL: Average chain length.

c Surface lipids include amphipathic phospholipids, lysophospholipids, sphingomyelin (SM), ceramides, free cholesterol, diacylglycerol, and monoacylglycerol. Core lipids include hydrophobic cholesteryl esters and triacylglycerol (TG).

d The mass spectrometry intensity was reported for APOA1 (apolipoprotein A-I), SAA1 (serum amyloid A-1), SAA2 (serum amyloid A-2), and APOL1 (apolipoprotein L-1).

e The adjusted model included mother’s baseline characteristics, including height, BMI, age, years of formal education, household food insecurity access score, asset index, housing index, malaria status, maternal alpha-1-acid glycoprotein (AGP) and C-reactive protein (CRP), and child’s sex.

f One-tailed test was performed.

g Two-tailed test was performed.

**Figure 1:**
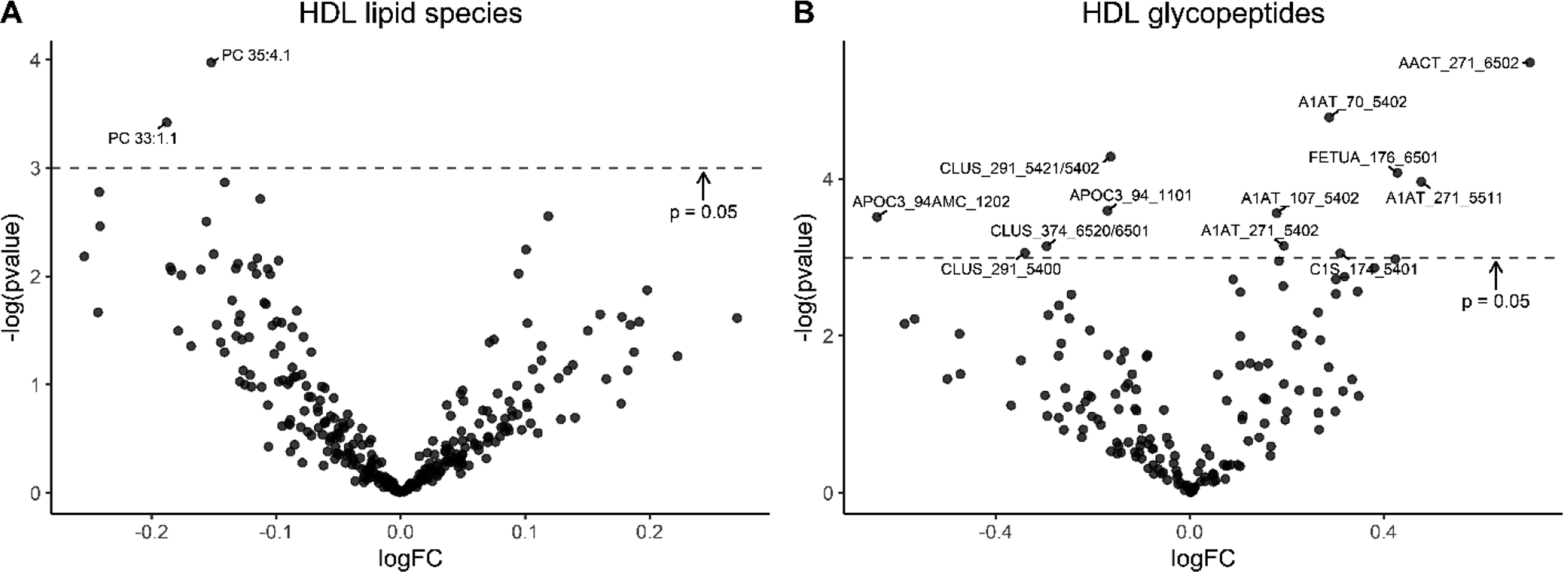
Volcano plots of the intervention effects on HDL lipid species (A) and HDL glycopeptides (B). The log fold changes of all measured variables are displayed on the *x*-axis and the −log(*p*-value) on the *y*-axis. Variables with *p*-value <0.05 were labeled. *p*-values were not corrected for multiple testing.

Three-hundred and thirteen lipid species from 12 lipid classes were quantified (Supporting Information Table S1). The effects of the intervention on lipid species are presented in the volcano plot of Figure 1A. Only two lipid species, phosphatidylcholine 35:4.1 (*p* = 0.0188) and phosphatidylcholine 33:1.1 (*p* = 0.033), were significantly different between intervention groups, and these differences did not remain statistically significant after correction for multiple testing (*p* = 0.998 and *p* = 0.998, respectively).

Thirty-three HDL-associated proteins and 163 glycopeptides from 21 proteins were quantified; the remaining 12 out of the 33 HDL-associated proteins did not contain glycopeptides (Supporting Information Tables S2 and S3). There were no statistically significant differences between intervention groups in any of the 33 HDL-associated proteins. The effects of the intervention on the glycopeptides are presented in the volcano plot of Figure 1B. The abundances of 12 glycopeptides differed between intervention groups. Four sialylated A1AT glycopeptides (i.e., A1AT_70_5402, A1AT_271_5511, A1AT_271_5402, and A1AT_107_5402) were higher in children in the SQ-LNS group (*p* = 0.008, *p* = 0.019, *p* = 0.043, and *p* = 0.028, respectively, before correction for multiple testing). The sialylated glycopeptides AACT_271_6502, FETUA_176_6501, and C1S_174_5401 were also higher in children given SQ-LNS (*p* = 0.004 and 0.017, and 0.047, respectively). Two APOC3 glycopeptides (APOC3_94AMC_1202 and APOC3_94_1101, *p* = 0.030 and 0.027, respectively) and three CLUS glycopeptides (CLUS_291_5421/5402, CLUS_374_6520/6501, and CLUS_291_5400, *p* = 0.014, *p* = 0.043, and *p* = 0.047, respectively) were lower in children given SQ-LNS. However, none of the group differences in these glycopeptides remained significant after correction for multiple testing.

**Figure 2:**
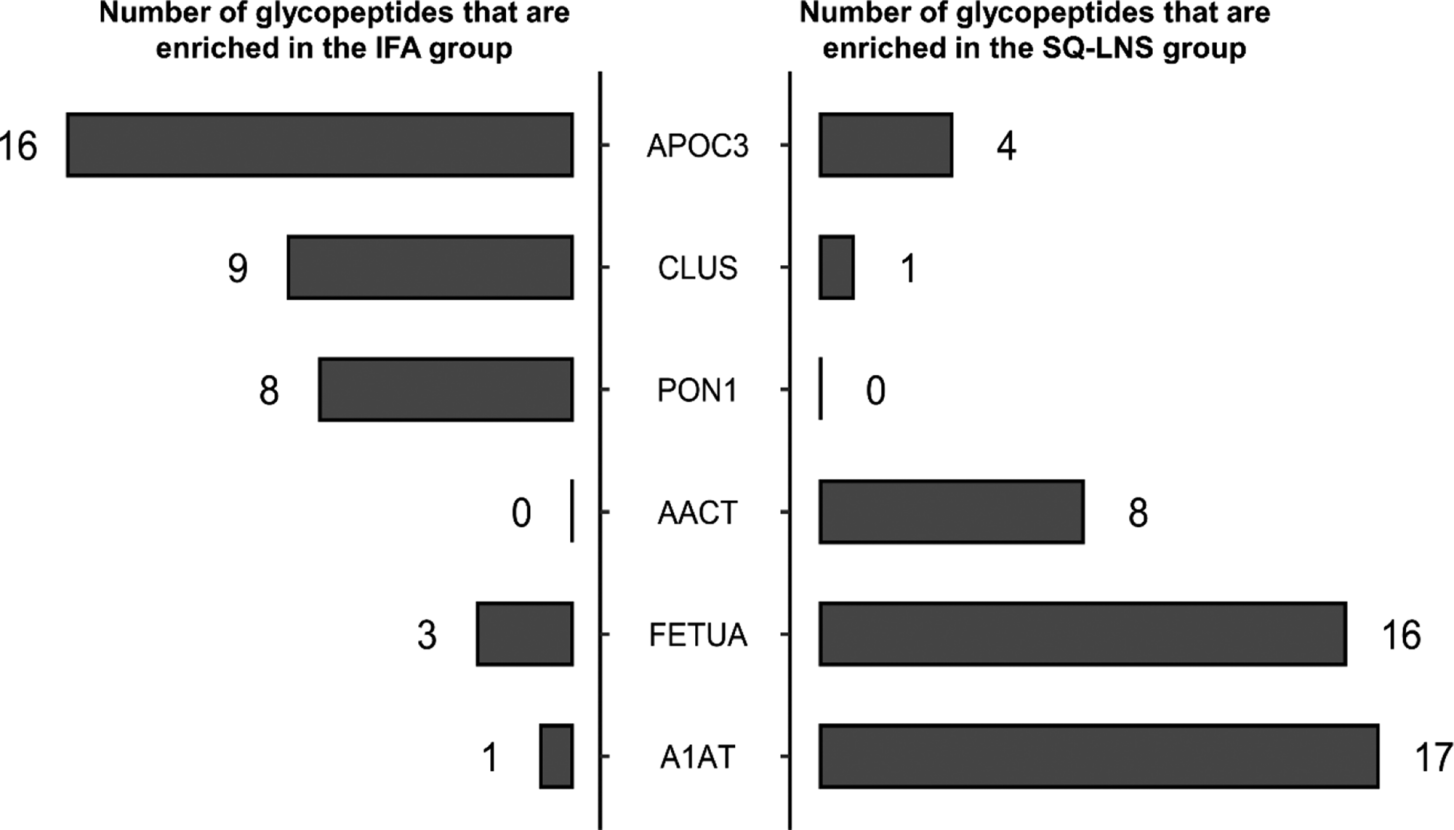
Enrichment analysis of glycopeptides in the IFA and SQ-LNS group. Out of the 33 HDL-associated proteins monitored, 21 contained glycopeptides. Six glycopeptides from a subset of the 21 proteins differed significantly in enrichment between intervention groups. Enrichment is characterized as the total amount of glycopeptides of a particular protein across all glycosylation sites as a measure of the degree of glycosylation of that protein. Number of glycopeptides of APOC3 (apolipoprotein C-III), CLUS (clusterin), PON1 (paraoxonase 1), AACT, FETUA, and A1AT (alpha-1-antitrypsin) that are lower (left panel) or higher (right panel) in children in the SQ-LNS compared to the IFA intervention group. SQ-LNS, small-quantity lipid-based nutrient supplements; IFA, iron and folic acid; HDL, high-density lipoproteins.

Enrichment analysis results for 21 proteins showed whether the glycopeptides of a particular protein were enriched within either group (Supporting Information Table S4). Glycopeptides of A1AT, fetuin A (FETUA), and alpha 1-antichymotrypsin (AACT) were significantly enriched in the SQ-LNS group (*p* < 0.001, *p* = 0.004, and *p* = 0.006, respectively), whereas glycopeptides of APOC3, CLUS, and PON1 were significantly enriched in the IFA group (*p* = 0.001, *p* = 0.004, and *p* = 0.002, respectively; Figure 2).

### Associations between Site-Specific Glycosylation and CEC

**Figure 3:**
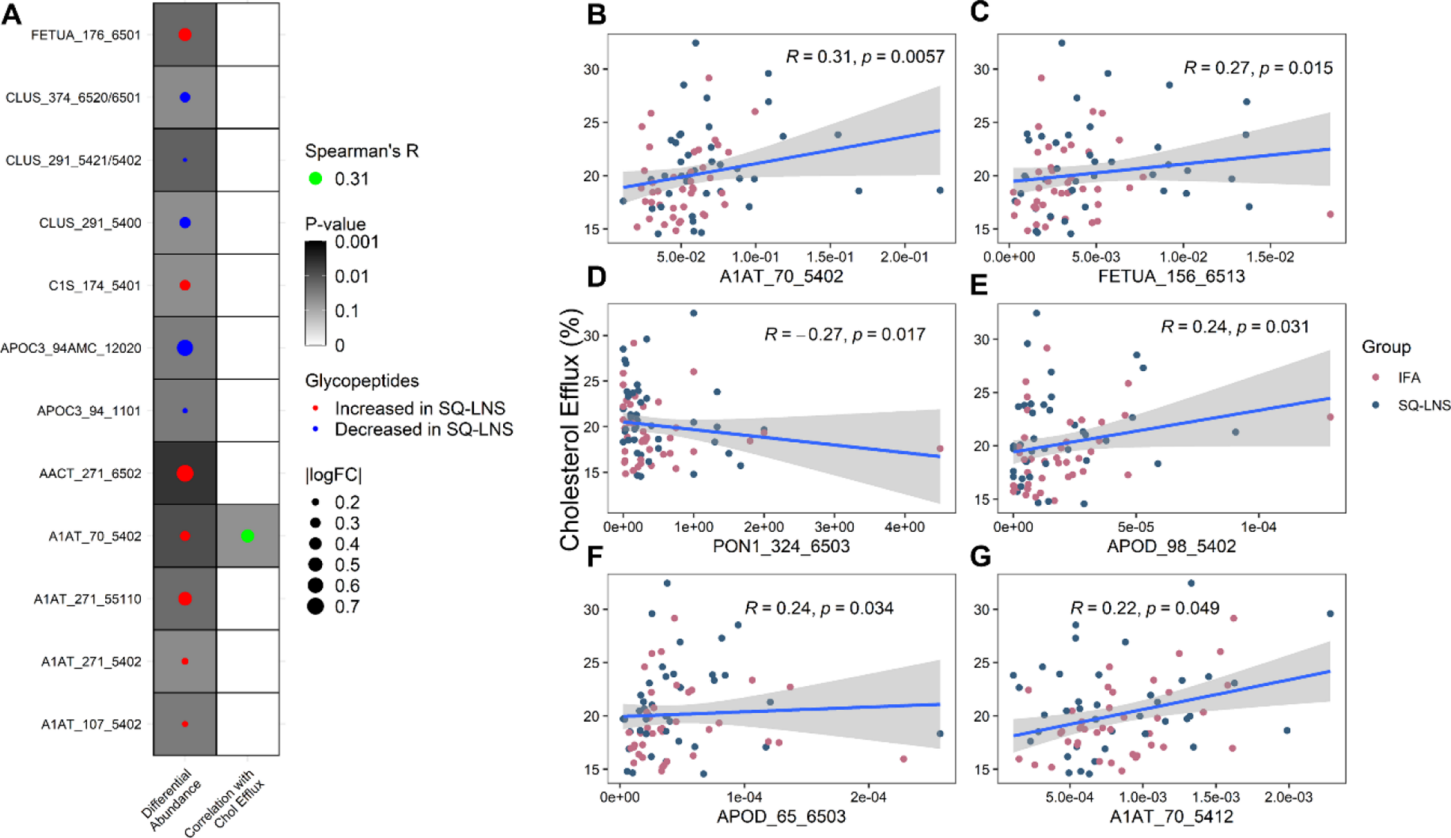
A: Dotmap of the SQ-LNS effects on HDL glycopeptides and glycopeptide correlation with CEC. Glycopeptides that were significantly different (*p* ≤ 0.05) between intervention groups are shown. The darkness of the background indicates the *p*-value. The dot size represents glycopeptide log fold changes in the abundance analysis. B–G: Scatterplot of all glycopeptides associated with HDL CEC, including glycopeptides A1AT_70_5402 (B), FETUA_156_6513 (C), PON1_324_6503 (D), APOD_98_5402 (E), APOD_65_6503 (F), and A1AT_70_5412 (G). CEC, cholesterol efflux capacity; HDL, high-density lipoprotein; SQ-LNS, small-quantity lipid-based nutrient supplements; A1AT, alpha-1-antitrypsin; FETUA, alpha-2-HS-glycoprotein; PON1, serum paraoxonase/arylesterase 1; APOD, apolipoprotein D.

Among the 12 glycopeptides that were altered by SQ-LNS intervention (Figure 3A), A1AT_70_5402 was also positively associated with HDL CEC (Spearman’s unadjusted *p* = 0.006, Figure 3B). Five additional glycopeptides (not altered by SQ-LNS) were significantly correlated with HDL CEC (Figure 3C–G): A1AT_70_5412 (unadjusted *p* = 0.049), FETUA_156_6513 (unadjusted *p* = 0.015), and two sialylated apolipoprotein D (APOD) glycopeptides, APOD_98_5402 and APOD_6503 (unadjusted *p* = 0.031 and *p* = 0.034, respectively), were positively associated with HDL CEC, whereas PON1_324_6503 was negatively associated (unadjusted *p* = 0.017) with HDL CEC. However, none of these associations remained significant after correction for multiple testing.

### Associations between the HDL Composition and Function and Growth Outcomes

We determined the associations between the primary HDL variables (HDL CEC, overall EOD_18_ and ACL, surface/core lipid ratio, HDL APOA1, SAA1, SAA2, and APOL1) and the growth outcomes (Supporting Information Table S5). Overall EOD_18_ was positively associated with the change in LAZ from 12 to 18 months in the adjusted model (*p* = 0.041). HDL APOA1 was positively associated with change in WAZ and WLZ from 12 to 18 months (*p* = 0.017 and *p* = 0.0105, respectively). HDL SAA1 and SAA2 were both positively associated with change in WAZ (*p* < 0.001 for both) and change in WLZ (*p* < 0.001 for both) from 12 to 18 months. APOL1 was positively associated with change in HCZ (*p* = 0.035), WAZ (*p* < 0.001), and WLZ (*p* < 0.001) from 12 to 18 months.

## Discussion

In this secondary outcome analysis of a subgroup of participants in the iLiNS-DYAD-Ghana study, we explored whether SQ-LNS given to both mothers and their children was related to child HDL composition and function at 18 months, and whether these HDL characteristics were associated with growth outcomes. The previously published results using the complete set of participants (*N* = 1228) demonstrated that children in the SQ-LNS group had significantly higher WAZ and LAZ at 18 months.^6^ In this study of a subset of 80 children, the results support our hypothesis that HDL from children in the SQ-LNS group had an increased capacity to efflux cholesterol from macrophage cells compared to HDL from children in the IFA group. We did not observe any significant differences in the level of HDL-associated proteins including APOA1, SAA, or APOL1, or their glycosylation compositions. However, there was a significant enrichment of glycopeptides in A1AT, FETUA, and AACT in the SQ-LNS group and an enrichment of glycopeptides in APOC3, CLUS, and PON1 in the IFA group. We also found that the HDL lipidome EOD_18_ was positively associated with the change in LAZ from 12 to 18 months, whereas the HDL-associated proteins including APOA1, SAA1, SAA2, and APOL1 were associated with other aspects of growth from 12 to 18 months.

To our knowledge, the HDL CEC of children in lower-income populations has not been previously studied. In Ireland, HDL CEC was negatively correlated with waist circumference and BMI in children at age 5 and 9 years, suggesting that overnutrition may have a detrimental effect on HDL cholesterol efflux.^23^ Among children under 6 years of age in India, decreased PON1 activity and antioxidant capacity were observed in 30 malnourished children compared to 30 healthy controls.^24^ Our results suggest that improved maternal and child nutrition may improve HDL CEC among children at 18 months of age. Cholesterol efflux is a critical function of HDL particles, regulating cellular cholesterol homeostasis and thus affecting a wide array of fundamental cellular activities.^25^ For example, by regulating cellular plasma membrane cholesterol content, HDL particles potentiate the innate immune response by increasing the ability of macrophages to clear respiratory tract bacterial infections.^14^ HDL particles also potentiate the adaptive immune response by regulating dendritic cell phenotype and activity.^26^

APOA1, as the defining HDL protein, is strongly linked with the ability of HDL particles to efflux cholesterol and is essential for the binding of HDL particles to the ABCA1 receptor.^27^ In this study, child HDL APOA1 concentration was not significantly higher in the SQ-LNS group, suggesting either that a larger sample size is needed to determine whether SQ-LNS increases HDL APOA1 or that another HDL parameter was responsible for the increase in efflux capacity.

The lack of effect of SQ-LNS on HDL lipidomic composition was somewhat surprising. We expected that providing SQ-LNS, which is enriched in fatty acids from soybean oil and peanut, would result in an enrichment of these fatty acids and a change in the overall saturation and chain length of fatty acids within HDL lipids. We expect that this lack of effect on the HDL lipidome may be due to the background diet and/or breastmilk fatty acid composition or that the amount of lipid in the supplement was less than what is needed to change the composition of HDL. EOD_18_ and ACL are estimations of the overall unsaturation and chain length, respectively, of fatty acids in the HDL lipidome. A higher EOD_18_ value means more unsaturated fatty acids in a sample, while a higher ACL means more long-chain fatty acids. The EOD_18_ and ACL observed in this sample of children at 18 months were 1.4 ± 0.1 double bonds and 15.6 ± 0.5 carbons, respectively. Breastmilk fatty acids tend to be enriched in medium-chain fatty acids (e.g., 12:0, 14:0, and 14:1), which may be contributing to the observed ACL in these children at 18 months of age, given that many of them were still breastfeeding.^28^ In fact, women in the SQ-LNS group had higher median ALA levels and ALA/arachidonic acid ratios in breast milk at 6 months postpartum compared to women in the MMN group (*p* = 0.02 and *p* = 0.02, respectively) and IFA group (nonsignificant for both), although these differences did not remain statistically significant after adjusted analysis.^29^

Proteins associated with HDL have different glycosylation patterns compared to the proteins in plasma, suggesting that glycosylation may be important for either directing proteins to HDL particles or conferring HDL-specific functions.^30^ We have also previously shown that the glycosylation profiles of APOC3, but not A1AT, FETUA, and apolipoprotein E (APOE), were changed in response to short-term diet change.^21^ However, the effects of longer-term diet change on HDL glycosylation profiles have not been previously reported. Our exploratory findings in this subset of 80 children suggest that SQ-LNS may alter the glycosylation of several key HDL-associated proteins. The abundances of 12 specific glycopeptides on six different HDL proteins were different in response to long-term provision of maternal and infant SQ-LNS. Seven of the glycopeptides that differed were derived from three acute-phase proteins (A1AT, AACT, and FETUA) and one immune protein (C1S), and these exhibited higher abundance in the SQ-LNS group. Moreover, A1AT, AACT, and FETUA, but not C1S, were found to be more highly glycosylated in the SQ-LNS group through enrichment analysis. Both A1AT and AACT are protease inhibitors released in response to a wide variety of inflammatory stimuli, to protect tissues from proteolytic degradation associated with the activity of immune cells, particularly neutrophils.^31,32^ FETUA is a lipid binding protein that has multiple roles, including its role as a negative acute-phase protein during sepsis and endotoxemia, its role in promoting wound healing, and its role in neuroprotection.^33,34^ C1S is a critical component in the activation of the classical complement pathway, which is involved in innate immunity.^35^ Five of the 12 glycopeptides that differed by intervention group were derived from two other HDL proteins, APOC3 and CLUS. For these, lower glycopeptide abundance and enrichment were observed in the SQ-LNS group. APOC3 is involved in the regulation of lipoprotein metabolism, and its concentration in preschool children born preterm has been found to be higher than in those born full term.^36^ CLUS is a glycoprotein with a multitude of functions including lipid transport and as a chaperone protein.^37^ In the enrichment analysis, PON1 glycosylation was also lower in the SQ-LNS group. PON1 is an enzyme that influences HDL antioxidant capacity, and its activity was found to be diminished in obese children compared with normal weight children.^38,39^ The implications of these glycoprofile changes associated with SQ-LNS in the context of growth and development in lower-income settings are currently unknown, and further work on their mechanism is needed.

Glycosylation involvement with HDL cholesterol efflux is largely unknown. Here, we found that six glycosylation sites on four HDL proteins were associated with CEC. Among these, two disialylated A1AT glycopeptides, A1AT_70_5402 and ATAT_70_5412, were positively associated with CEC. Interestingly, both of these glycopeptides have been previously reported to be associated with CEC in healthy adults.^21^ Thus, the site-specific glycosylation of A1AT may play a role in modulating HDL functional capacity, but the underlying mechanism by which glycosylation alters HDL function remains to be explored.

The HDL lipid EOD_18_ was significantly correlated with change in LAZ from 12 to 18 months in this study, which suggests that higher levels of fatty acid unsaturation or higher dietary intake of unsaturated fatty acids may have beneficial effects on linear growth. Indeed, a cross-sectional study in Malawi found that stunted young children aged 12 to 59 months had lower levels of serum ω-3 and ω-6 polyunsaturated fatty acids.^40^ We found that HDL APOA1 was positively associated with WAZ and WLZ changes from 12 to 18 months, which suggests that a higher HDL APOA1 content during early development may be associated with increased ponderal growth. However, these results may not be generalizable to populations of different geographical regions. For example, the iLiNS-DYAD trial in Malawi showed no changes in linear growth in the SQ-LNS group compared with the IFA group at 18 months of age.^41^

The strengths of this study include a detailed analysis of HDL composition and in vitro assessment of HDL functional capacity in a well-characterized cohort of young children. The subcohort of 80 children selected for this study was similar to the whole cohort (*n* = 440 for IFA and *n* = 441 for SQ-LNS) from the larger iLiNS-DYAD-Ghana study with respect to the mean and standard deviation of the WAZ, LAZ, HCZ, and WLZ scores.^6^ The exploratory nature of the study and the small sample size are limitations of the study. While the aim of this pilot study is to investigate the function and composition of HDL particles in children, further research should explore whether the composition of their low-density lipoprotein (LDL) particles is altered by SQ-LNS.

In this sample of children, we demonstrated that provision of SQ-LNS had a beneficial effect on HDL CEC and potentially also on HDL protein glycosylation, but not lipidomic composition or protein abundance in HDL. Further research is needed to evaluate whether an improvement in CEC because of SQ-LNS is evident in other populations and to investigate the long-term impact of improved HDL functional capacity on the growth and development of young children.

## Experimental Procedures

### Samples and Subjects

The complete study design and subject characteristics of the iLiNS DYAD-Ghana trial were described in detail previously.^5^ The compositions of IFA and SQ-LNS are presented in Supporting Information Table S6. In total, 1320 women were enrolled at a mean gestational age of 16.3 weeks and were randomized to receive IFA, MMN, or SQ-LNS until delivery followed by placebo (200 mg calcium), MMN, or SQ-LNS, respectively during 6 months postpartum. The infants whose mothers were supplemented with SQ-LNS received SQ-LNS formulated for infants from 6 to 18 months of age; children in the IFA group did not receive supplements. Women were visited biweekly during pregnancy and weekly after birth to deliver fresh supplies of supplements and monitor supplement consumption and morbidity. Blood was drawn from children at 18 months at the laboratory, and plasma was separated after centrifugation at 1252 g for 15 min at room temperature. Plasma samples were stored at −33 °C in Ghana before they were airmailed on dry ice to Davis, CA, USA, after which time the samples were stored at −80 °C.^42^

In this study, a subset of 40 children from the IFA group and 40 from the SQ-LNS group were selected, and their plasma samples at 18 months were analyzed. The 80 children were selected according to the following maternal criteria: (1) randomized to either SQ-LNS or IFA (the MMN group was excluded); (2) primiparous; (3) not overweight (BMI < 25 kg/m^2^ at enrollment); and (4) enrolled between October 2010 and December 2011 (to avoid inclusion of women enrolled earlier who may have had mixed exposure to IFA and MMN, as explained previously).^5^ The maternal criteria were selected to maximize the probability of observing a response to SQ-LNS, as the effects of SQ-LNS on birth size were greater among infants born to primiparous mothers,^5^ and longer-term effects on child growth were greater among children born to nonoverweight women.^43^ In total, 116 children met these criteria, 53 in the SQ-LNS group and 63 in the IFA group. These children were randomly sorted, and the first 40 in each group were selected (Supporting Information Figure S1).

The study protocol was approved by the ethics committees of the University of California, Davis; the Ghana Health Service; and the University of Ghana Noguchi Memorial Institute for Medical Research and was registered on clinicaltrials.gov as NCT00970866.

### HDL Isolation

HDL particles were isolated through a two-step HDL isolation method modified from the study by Holzer et al.^44^ which isolates HDL particles first by density using sequential flotation ultracentrifugation as previously described^45^ followed by fast protein liquid chromatography (FPLC). Briefly, 500 μL of plasma was underlaid under KBr solution at a density of 1.006 g/mL to remove triglyceride-rich, low-density (<1.006 g/mL) particles, including chylomicrons and very low-density lipoproteins, and subjected to ultracentrifugation in an Optima MAX-TL Ultracentrifuge with (Beckmann-Coulter) fixed angle rotor at 110,000 rpm and 14 °C for 30 min. After centrifugation, the supernatant was removed by aspiration, and the remaining fraction containing HDL, LDL, albumin, and plasma proteins was adjusted to a density of 1.210 g/mL with 1.340 g/mL KBr solution and underlaid under clean 1.210 g/mL density solution and then subjected to ultracentrifugation at 110,000 rpm and 14 °C for 3 h and 30 min. The supernatant was removed by aspiration and dialyzed using an Amicon Ultra-4 50 kDa centrifugal filter (Millipore) by centrifugation at 4500 rpm for 8 min. A final volume of 250 μL was then transferred to an amber vial for FPLC analysis using a single Superdex 200 Increase 10/300 GL agarose-crosslinked column (GE Healthcare) on an AKTA P-920 FPLC (Amersham Biosciences) connected to a fraction collector. Four 1 mL fractions eluting in the HDL size range were pooled together and dialyzed to 100 μL of which one aliquot was used for glycoproteomic analysis, another for lipidomics analysis, and one for analysis of CEC.

### HDL Protein and Glycoprotein Identification

Isolated HDL samples were run on an Agilent 1290 Infinity II high-performance liquid chromatograph coupled to a Fusion Lumos MS/MS Orbitrap (Thermo Fisher Scientific). Peptides and glycopeptides were identified with Byonic software (Protein Metrics Inc) from the Orbitrap MS data (see Supporting Information Material 1 for additional details).

A total of 33 HDL-associated proteins were monitored in this study including apolipoprotein A-I (APOA1), apolipoprotein(a) (LPA), apolipoprotein A-II (APOA2), apolipoprotein A-IV (APOA4), apolipoprotein A-V (APOA5), apolipoprotein C-II (APOC2), apolipoprotein C-IV (APOC4), apolipoprotein F, apolipoprotein L1 (APOL1), haptoglobin-related protein, phosphatidylcholine-sterol acyltransferase, phospholipid transfer protein, alpha-1-antitrypsin (A1AT), alpha-1B-glycoprotein (A1BG), alpha-1-antichymotrypsin (AACT), apolipoprotein B-100 (APOB100), apolipoprotein C-I (APOC1), apolipoprotein C-III (APOC3), APOD, APOE, beta-2-glycoprotein 1 (APOH), apolipoprotein M (APOM), complement C1s subcomponent (C1S), clusterin (CLUS or APOJ), complement C3 (C3), alpha-2-HS-glycoprotein (FETUA or AHSG), hemopexin (HPX), heparin cofactor 2 (HCF2), kininogen-1 (KNG1), serum paraoxonase/arylesterase 1 (PON1), serum amyloid A-4 (SAA4), serum amyloid A-1 (SAA1), and serum amyloid A-2 (SAA2).

### Targeted Glycoproteomics Analysis

(Glyco)peptides were quantified on an Agilent 1290 Infinity II LC system coupled to an Agilent 6495B Triple Quadrupole MS. A commercially available human serum (Sigma-Aldrich) was also digested to serve as sample preparation controls. Protein standards (APOA1, APOC1, APOD, APOE, and CLUS; all from Sigma-Aldrich) were mixed and digested with the batch to serve as calibration standards (see Supporting Information Material 2 for additional details).

A transition list for target analytes was created by combining previously reported transitions^30,46^ with new transitions selected from the Orbitrap analysis. The transition list included 47 peptides and 163 glycopeptides from 33 proteins. The instrument was run on dynamic multiple reaction monitoring mode to minimize the number of transitions being monitored at each scan cycle. For peptides, at least two product ions were selected for monitoring. Quantitation was based on the area of the more abundant product ion while the other ions monitored were for qualitative identification. Abundance is the amount of a glycopeptide in ion counts normalized to the ion counts of the nonglycosylated peptide, which is used as a measure of the total amount of that protein. Product ions for glycopeptides were based on diagnostic glycan fragments.

### HDL Lipidomic Analysis

The HDL lipidomic profile was measured at the West Coast Metabolomics Center, using a previously reported protocol.^47^ Briefly, 225 μL of cold internal standard mixture was added into 25 μL purified HDL sample followed by adding 750 μL cold methyl *tert*-butyl ether containing cholesteryl ester (CE) 22:1, and 188 μL of distilled water was added after shaking at 4 °C for 6 min. Following centrifugation at 14,000 ×*g* for 2 min, 350 μL supernatant was extracted, dried down, and resuspended with 65 μL methanol/toluene (9:1, v/v) solution, and 3 μL of the resuspended sample was then injected into a LCMS for analysis. Each sample was injected in parallel into an Agilent 6530b quadrupole time-of-flight (QTOF) and a 6550 QTOF for positive and negative modes respectively to capture as many complex lipid species as possible. Liquid chromatography separation was performed on a Waters Ultra-Performance Liquid Chromatography CSH C18 column (1.7 μM, 2.1 mm 100 mm), using a gradient method. A quality control (QC) sample was run every 11^th^ injection. The QC samples all came from the same human plasma pool. The internal standard mixture contained ceramide (Cer) d18:1/17:0, d7-cholesterol, diacylglycerol 12:0/12:0, lysophosphatidylcholine 17:0, lysophosphatidylethanolamine 17:1, monoacylglycerol 17:0, phosphatidylcholine 12:0/13:0, phosphatidylethanolamine 17:0/17:0, SM d18:1/17:0, sphingosine (d17:1), and *d*_5_-TG 17:0/17:1/17:0 dissolved in methanol. Each lipid species was calibrated to the internal standard which belongs to the same lipid class. The concentration of lipid classes was calculated by aggregating all lipid species which belong to the same lipid class. Lipidomics summarized variables including equivalent of double-bound per 18 carbons (EOD_18_), ACL, and surface/core lipid ratios were calculated as reported previously.^18^

### Analysis of CEC

HDL CEC was measured using a commercially available kit (Abcam, ab19685) using a protocol as reported previously.^48^ J774A.1 (ATCC, TIB-67) macrophage cells were first cultured for 4 h in Roswell Park Memorial Institute 1640 medium with 10% fetal bovine serum and fluorescently labeled with cholesterol labeling reagent for another 4 h. Cells were then washed and incubated for 2 h with isolated HDL fractions, together with acyl-CoA/cholesterol acyltransferase inhibitors and 3′,5′-cyclic adenosine monophosphate. The cellular supernatant was removed, and cells were lysed using MPER cell lysis buffer (Thermo Scientific, 78501). The fluorescence intensity in the supernatant and cells was measured separately using a Synergy H1 plate reader (BioTek). The cellular cholesterol capacity was calculated as follows:
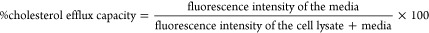


### Statistical Analysis

The statistical analysis plan was posted before analysis (https://ilins.ucdavis.edu/). With our sample size of 40 per group, we can detect an effect size of 0.64 SD in the mean difference between groups, assuming an alpha of 0.05 and 80% power. Data were analyzed on an intention-to-treat basis whereby children were included regardless of adherence to the intervention. Data analysis was performed in R (3.6.1). The distributions of outcome variables and key baseline variables were inspected for normality using the Shapiro–Wilks test. A Shapiro–Wilks statistic larger than 0.95 was considered normally distributed. Outcome variables were transformed as necessary with a natural log transformation, and if this still did not normalize the distribution, normalized ranks or categories were created. Maternal background characteristics were summarized as mean ± SD according to their intervention group assignment at enrollment (IFA or SQ-LNS). The household assets index, housing index, and household food insecurity access score were calculated as proxy indicators for women’s socioeconomic status as reported previously.^5^ The children’s growth status was expressed as weight for age z-score (WAZ), length for age z-score (LAZ), weight for length z-score (WLZ), and head circumference z-score (HCZ), calculated according to the World Health Organization standard.^49^

Unadjusted and adjusted linear models were used to test the impact of the intervention group (SQ-LNS vs IFA) on the HDL lipidome, proteome, and CEC. The adjusted model included the mother’s baseline characteristics, including height, BMI, age, years of formal education, household food insecurity access score, asset index, housing index, malaria status, maternal AGP and CRP, and child’s sex. A covariate was included in the adjusted model if it was correlated with the outcome variable using Pearson’s correlation test (*p* < 0.1). We hypothesized that SQ-LNS provided to both mothers and their children would increase child CEC; we conducted a one-tailed test for significance because it is very unlikely that SQ-LNS could cause a decrease in this outcome, given that SQ-LNS contain ALA, which has been shown to increase CEC in vitro.^22^ Two-tailed tests were performed for the lipidomics primary outcomes, HDL EOD_18_, ACL, and surface/core lipid ratio, and for the proteomics primary outcomes, HDL APOA1, SAA1, SAA2, and APOL1 level. We hypothesized that because the SQ-LNS supplement provided essential fatty acids, the average desaturation and chain length of the fatty acids within HDL particles and the ratio of surface lipids (i.e., phospholipids) to core lipids (i.e., cholesterol esters) would be altered among children in the SQ-LNS group. We also hypothesized that the content of the main apolipoprotein associated with HDL and APOA1, as well as the content of proteins linked to immune activation and inflammation (i.e., SAA1, SAA2, and APOL1), would be altered in the SQ-LNS group.

An exploratory analysis was performed to examine whether the intervention group was related to the secondary outcomes, HDL lipidome species or glycopeptides, using linear models with two-tailed tests. In the exploratory analysis, a Benjamini–Hochberg test was performed to correct for multiple testing. Enrichment analysis was performed using the phyper function in R’s stats package to test whether the glycopeptides of each protein were enriched in either intervention groups. Enrichment is characterized as the total amount of glycopeptides of a particular protein across all glycosylation sites as a measure of the degree of glycosylation of that protein. The glycan signal nomenclature follows the conventions of the Consortium for Functional Glycomics. The glycopeptides are labeled as Protein_Position_GlycanComposition_ChargeState. Glycan compositions are written as four-digit numbers indicating the number of hexose (mannose or galactose), *N*-acetylhexosamine (*N*-acetylgalactosamine), fucose, *N*-acetylneuraminic acid, or sialic acid (Neu5Ac), respectively. For example, A1AT_70_5402 represents the glycopeptide of A1AT consisting of 5 hexose, 4 *N*-acetylhexosamine, 0 fucose, and 2 Neu5Ac.

An additional exploratory analysis was performed to examine the association between HDL glycosylation and CEC to identify which compositional changes to the HDL particles were associated with improvement in cholesterol efflux. We have previously found that glycoprofiles of HDL-associated proteins were highly correlated with CEC.^21^ For the association between CEC and HDL glycosylation, a Spearman’s test was used to reduce the effect of outliers.

We also explored the associations between HDL variables (lipidome, proteome, and CEC) and growth outcomes, including growth status at 18 months (WAZ, LAZ, WLZ, and HCZ) and change in WAZ, LAZ, WLZ, and HCZ from 12 to 18 months, using both unadjusted and adjusted models. The potential covariates in the adjusted models included maternal age, height, BMI, malaria status, hemoglobin, asset, and housing index, household food insecurity access score, and years of formal education, as well as child morbidity (number of episodes of respiratory infections, fever, loose stool, and poor appetite from 6 to 18 months). Covariates were included in the adjusted model if they were correlated with the growth outcome using Pearson’s correlation test (*p* < 0.1).

## Supporting Information Available

Flowchart of the study profile (Supporting Information Figure S1), detailed experimental procedures for HDL protein and glycoprotein identification and target glycoproteomic analysis (Supporting Information Material 1–2), and supplemental tables for lipid species relative abundance, HDL-associated proteins mean abundance, glycopeptide analysis, enrichment analysis, and supplement composition (Supporting Information Tables S1–6) (PDF)

## Abbreviations

A1AT: alpha-1-antitrypsin
APOA-I: apolipoprotein A-I
APOL1: apolipoprotein L1
ACL: average chain length
BMI: body mass index
CEC: cholesterol efflux capacity
CLUS: clusterin or APOJ
EOD_18_: equivalent of double-bond per 18 carbons
HCZ: head circumference for age z-score
HDL: high-density lipoproteins
IFA: iron and folic acid
iLiNS: International Lipid-Based Nutrient Supplements
LAZ: length for age z-score
SAA1: serum amyloid A-1
SAA2: serum amyloid A-2
SQ-LNS: small-quantity lipid-based nutrient supplements
WAZ: weight for age z-score
