# Supplementary material for: Lipid-Based Nutrient Supplementation Increases High-Density Lipoprotein (HDL) Cholesterol Efflux Capacity and Is Associated with Changes in the HDL Glycoproteome in Children
Source: ACS Omega. 2021 Nov 18;6(47):32022–31. doi: 10.1021/acsomega.1c04811 (PMC8638293; doi:10.1021/acsomega.1c04811)
Supplement: Supplementary file 1 — ao1c04811_si_001.pdf [file ao1c04811_si_001.pdf]

Lipid-Based Nutrient Supplementation Increases HDL Cholesterol Efflux Capacity and is Associated with Changes in the HDL Glycoproteome in Children

Brian V. Hong[a], Chenghao Zhu[a], Maurice Wong[b], Romina Sacchi[a], Christopher H. Rhodes[a], Jea Woo Kang[a], Charles D. Arnold[a], Seth Adu-Afarwuah[c], Anna Lartey[c], Brietta M. Oaks[d], Carlito B. Lebrilla[b], Kathryn G. Dewey[a], Angela M. Zivkovic[a*]

[a]Department of Nutrition and [b]Chemistry, University of California, Davis, Davis, CA, USA; [c]Department of Nutrition and Food Science, University of Ghana, Legon, Ghana; [d]Department of Nutrition and Food Sciences, University of Rhode Island, Kingston, RI, USA

[*] To whom correspondence should be addressed.

E-mail: amzivkovic@ucdavis.edu

Online Supporting Information: Tables S1-6, Figure S1, Method S1-2

**Table S1**. Lipid species relative mean abundance.			
Treatment	Lipid Species (n = 313)	Mean (%)	SD
IFA	CE 16:1	0.1378572	0.0604765
IFA	CE 18:1	2.6030622	0.75991
IFA	CE 18:2	16.2803028	3.5456791
IFA	CE 18:3	0.1464146	0.0369396
IFA	CE 20:3	0.2330877	0.0856682
IFA	CE 20:4	1.8760629	0.6399473
IFA	CE 20:5	0.387712	0.2444259
IFA	CE 22:6	0.6374623	0.1987288
IFA	Ceramide 42:2 d	0.0043721	0.0018045
IFA	Cholesterol	7.853734	1.2850934
IFA	DG 32:1	0.0345559	0.0189325
IFA	DG 34:1	0.1713322	0.0858138
IFA	DG 34:2	0.1330312	0.0666908
IFA	DG 34:3	0.0088923	0.0065974
IFA	DG 36:1	0.0247751	0.0138689
IFA	DG 36:2	0.1829097	0.0884051
IFA	DG 36:3	0.2134531	0.0900126
IFA	DG 36:4 A	0.0370132	0.0209697
IFA	DG 38:5	0.0368875	0.0142032
IFA	DG 38:6	0.0246981	0.0115721
IFA	GlcCer 40:1 d	0.0058233	0.0016308
IFA	GlcCer 42:1 d	0.0099992	0.0024896
IFA	GlcCer 42:2 d	0.0102188	0.0031231
IFA	Gal-Gal-Cer 42:2 d	0.0009613	0.0003257
IFA	LPC 20:0	0.0017111	0.0010692
IFA	LPC 20:1	0.0010695	0.000808
IFA	LPC 22:4	0.0013012	0.0009683
IFA	LPC 22:6	0.0027707	0.0016736
IFA	LPC 16:0 o	0.0044347	0.0043819
IFA	LPC 16:0 p	0.0030814	0.0023026
IFA	LPC 16:0	0.0521558	0.0339188
IFA	LPC 18:0	0.0933325	0.0812941
IFA	LPC 18:2	0.004914	0.002191
IFA	LPC 20:3	0.0008577	0.0005119
IFA	LPC 20:4	0.0038756	0.0017815
IFA	LPC 20:5	0.0005545	0.0004261
IFA	PC 25:0	0.0056509	0.003086
IFA	PC 37:3	0.0070631	0.0020255
IFA	PC 39:6	0.0186046	0.0098897
IFA	PC 42:5	0.0032337	0.0012247

IFA	PC 42:6	0.001678	0.0011308
IFA	PC 32:0 p	0.0331638	0.0085406
IFA	PC 36:3 p	0.2746408	0.0721579
IFA	PC 38:2 p	0.0073635	0.0019078
IFA	PC 42:3 p	0.0074855	0.0017851
IFA	PC 34:1	0.0625206	0.0131247
IFA	PC 36:3	0.0763789	0.0168516
IFA	PC 36:5	0.631321	0.1060358
IFA	PC 38:4	0.035043	0.012812
IFA	PC 40:6	0.0257932	0.0092326
IFA	PC 28:0	0.0052521	0.0039004
IFA	PC 30:0	0.1326906	0.0752787
IFA	PC 31:0	0.0031081	0.001485
IFA	PC 32:0	0.1684637	0.0460285
IFA	PC 32:1	0.1883644	0.1053984
IFA	PC 33:0	0.0048116	0.0019503
IFA	PC 33:1	0.026403	0.0113446
IFA	PC 33:2	0.0258754	0.0093034
IFA	PC 34:0	0.0315977	0.0103688
IFA	PC 34:1.1	5.8217695	1.7982886
IFA	PC 34:2	12.73141	1.8240495
IFA	PC 34:3	0.02341	0.0157101
IFA	PC 35:1	0.0265742	0.0080173
IFA	PC 35:2	0.0621283	0.0299071
IFA	PC 35:3	0.0155381	0.0065739
IFA	PC 35:4	0.0072446	0.0019098
IFA	PC 36:1	0.6186497	0.2206464
IFA	PC 36:2	5.9438642	1.4155724
IFA	PC 36:3.1	1.0766842	0.3876907
IFA	PC 36:3.2	1.145882	0.3222141
IFA	PC 36:4	0.2463351	0.1274005
IFA	PC 36:4.1	0.0833027	0.0404342
IFA	PC 36:5.1	0.0103022	0.0179414
IFA	PC 36:6	0.0132486	0.0075397
IFA	PC 37:4	0.0189128	0.0105022
IFA	PC 37:6	0.0065841	0.0021936
IFA	PC 38:2	0.0401641	0.0088659
IFA	PC 38:3	0.3974327	0.1247121
IFA	PC 38:4.1	0.0863833	0.0339879
IFA	PC 38:5	0.6184815	0.167185
IFA	PC 38:5.1	0.2949042	0.1473133
IFA	PC 38:6	7.1234569	1.5630188
IFA	PC 38:7	0.0066066	0.0029699

IFA	PC 40:4	0.0253843	0.0090688
IFA	PC 40:5	0.2499824	0.0757798
IFA	PC 40:5.1	0.0333164	0.0131165
IFA	PC 40:6.1	1.1147742	0.3396461
IFA	PC 40:7	0.0089394	0.0311764
IFA	PC 40:8	0.0146751	0.0043687
IFA	PC 32:0 o	0.0275527	0.0086761
IFA	PC 34:0 o	0.0027981	0.0009148
IFA	PC 32:1 p	0.0061988	0.0024647
IFA	PC 34:1 p	0.0788858	0.0274006
IFA	PC 34:2 p	0.1204576	0.0295573
IFA	PC 36:1 p	0.0041282	0.0010508
IFA	PC 36:2 p	0.0153018	0.0035698
IFA	PC 36:4 p	0.3511014	0.106525
IFA	PC 38:3 p	0.081097	0.0204898
IFA	PC 38:4 p	0.2806301	0.0864274
IFA	PC 38:4 p.1	0.0503431	0.0153801
IFA	PC 38:5 p	0.1342734	0.0422402
IFA	PC 40:3 p	0.0121976	0.0030706
IFA	PC 40:4 p	0.0214711	0.0055188
IFA	PC 40:5 p	0.0368656	0.0113642
IFA	PC 40:6 p	0.0971024	0.0354183
IFA	PC 40:6 p.1	0.0334665	0.009923
IFA	PC 42:5 p	0.0065463	0.0025268
IFA	PE 34:3	0.1146389	0.0599862
IFA	PE 38:7	0.3375418	0.1415107
IFA	PE 34:2	0.0919267	0.0620603
IFA	PE 36:1	0.0300185	0.0232051
IFA	PE 36:4	0.0843725	0.0622276
IFA	PE 38:6	0.2139939	0.1331927
IFA	PE 34:1 p	0.0425784	0.0184177
IFA	PE 36:2 p	0.1046856	0.0617247
IFA	PE 36:4 p	0.3991413	0.1958227
IFA	PE 38:4 p	0.3761954	0.1745742
IFA	PE 38:5 p	0.1951929	0.0875122
IFA	PE 40:5 p	0.0485444	0.0215723
IFA	SM 37:1 d	0.007616	0.0023158
IFA	SM 38:0 d	0.0088763	0.0060071
IFA	SM 39:2 d	0.0052143	0.0014382
IFA	SM 42:0 d	0.0047196	0.0027203
IFA	SM 44:2 d	0.004052	0.0021327
IFA	SM 42:1 d	0.0310399	0.0213524
IFA	SM 30:1 d	0.0069589	0.0043363

IFA	SM 32:0 d	0.0039972	0.0028345
IFA	SM 32:2 d	0.0097454	0.0048926
IFA	SM 34:0 d	0.0624555	0.0215191
IFA	SM 34:2 d	0.1605549	0.0327618
IFA	SM 36:0 d	0.0315585	0.0254066
IFA	SM 36:1 d	0.2317237	0.0722986
IFA	SM 36:2 d	0.2964634	0.0623167
IFA	SM 36:3 d	0.0037257	0.0012834
IFA	SM 38:1 d	0.1294476	0.0353464
IFA	SM 38:2 d	0.0740422	0.0151675
IFA	SM 40:0 d	0.0128439	0.0073279
IFA	SM 40:1 d	0.2706064	0.06438
IFA	SM 40:2 d	0.139354	0.0274346
IFA	SM 42:2 d	0.6394989	0.2165979
IFA	SM 42:3 d	0.3308828	0.0801847
IFA	SM 43:1 d	0.0017848	0.0010368
IFA	TG 52:4	0.0332697	0.0144661
IFA	TG 40:0	0.007016	0.0054923
IFA	TG 42:0	0.0106121	0.0095075
IFA	TG 42:1	0.0060958	0.0056794
IFA	TG 42:2	0.0046848	0.0032918
IFA	TG 42:3	0.0038745	0.0035986
IFA	TG 44:0	0.0256535	0.0191692
IFA	TG 44:1	0.0240785	0.0266836
IFA	TG 46:0	0.1032078	0.0600261
IFA	TG 46:1	0.0809097	0.0676569
IFA	TG 46:2	0.0360252	0.0316921
IFA	TG 46:3	0.0025536	0.0016564
IFA	TG 48:1	0.5659861	0.3226701
IFA	TG 48:2	0.1440778	0.0914472
IFA	TG 48:3	0.0408615	0.0288546
IFA	TG 48:4	0.0066347	0.0055146
IFA	TG 49:1	0.0041158	0.0020547
IFA	TG 49:2	0.0158728	0.0073096
IFA	TG 49:3	0.0033593	0.0016306
IFA	TG 50:0	0.0590218	0.0302177
IFA	TG 50:1	1.0021554	1.0765837
IFA	TG 50:2	1.2092873	0.5671286
IFA	TG 50:3	0.2525405	0.14027
IFA	TG 50:4	0.0419162	0.0265651
IFA	TG 50:5	0.0039582	0.0024576
IFA	TG 51:1	0.019165	0.0089742
IFA	TG 51:2	0.0363267	0.018948

IFA	TG 51:3	0.0186234	0.0094593
IFA	TG 51:4	0.0035598	0.0020191
IFA	TG 52:1	0.2406567	0.1373493
IFA	TG 52:2	2.1983564	1.5269972
IFA	TG 52:3	2.592215	1.1982277
IFA	TG 52:4.1	0.6601893	0.3405633
IFA	TG 52:5	0.0345012	0.0222165
IFA	TG 52:6	0.0066296	0.0051064
IFA	TG 53:1	0.0070831	0.0026819
IFA	TG 53:2	0.0217316	0.0121459
IFA	TG 53:3	0.0211441	0.0102973
IFA	TG 53:4	0.0105091	0.0044903
IFA	TG 54:1	0.0278167	0.0144222
IFA	TG 54:2	0.1824766	0.1045102
IFA	TG 54:3	0.7642644	0.4227972
IFA	TG 54:4	0.525667	0.3337828
IFA	TG 54:5	0.1624144	0.0669403
IFA	TG 54:6	0.0637448	0.0338624
IFA	TG 56:3	0.0116036	0.0062336
IFA	TG 56:4	0.0168888	0.0131314
IFA	TG 56:5	0.0390634	0.0200898
IFA	TG 56:6	0.1072349	0.0411735
IFA	TG 56:7	0.1827324	0.1075734
IFA	TG 56:8	0.0016791	0.0014794
IFA	TG 57:1	0.0083727	0.0071137
IFA	TG 58:1	0.004774	0.003442
IFA	TG 58:3	0.0030075	0.0020274
IFA	TG 58:6	0.0055418	0.0025839
IFA	TG 58:8	0.0047328	0.0036193
IFA	TG 58:9	0.0309698	0.0200027
IFA	Ceramide 32:1 d	0.0002928	0.0001133
IFA	Ceramide 34:0 d	0.0006396	0.0002646
IFA	Ceramide 34:1 d	0.0027725	0.0008183
IFA	Ceramide 34:2 d	0.0002763	0.0000688
IFA	Ceramide 36:1 d	0.001005	0.0004663
IFA	Ceramide 38:1 d	0.000695	0.0002562
IFA	Ceramide 39:1 d	0.0002916	0.0001141
IFA	Ceramide 40:0 d	0.0012619	0.0005959
IFA	Ceramide 40:1 d	0.0033829	0.0009764
IFA	Ceramide 40:2 d	0.0004708	0.0001347
IFA	Ceramide 41:1 d	0.0024802	0.0007011
IFA	Ceramide 42:0 d	0.0019383	0.0009137
IFA	Ceramide 42:1 d	0.0159552	0.0060927

IFA	Ceramide 44:1 d	0.0002671	0.0001076
IFA	FA 12:0	0.0066281	0.0071516
IFA	FA 13:0	0.0009401	0.0004714
IFA	FA 14:0	0.0287537	0.0217693
IFA	FA 14:1	0.0010194	0.0006833
IFA	FA 15:0	0.0161904	0.0085919
IFA	FA 15:1	0.0016708	0.0010802
IFA	FA 16:0	1.9198203	1.4006561
IFA	FA 16:1	0.0191451	0.0123539
IFA	FA 17:0	0.0247965	0.014157
IFA	FA 18:1	0.1646577	0.1001472
IFA	FA 18:2	0.0313803	0.015915
IFA	FA 18:3	0.0009323	0.0004946
IFA	FA 20:0	0.0533295	0.0344251
IFA	FA 20:1	0.0057359	0.0027089
IFA	FA 20:2	0.0011786	0.0008093
IFA	FA 20:3	0.0003761	0.0002074
IFA	FA 20:3.1	0.0007403	0.000316
IFA	FA 20:4	0.0023513	0.0008496
IFA	FA 20:5	0.0007189	0.0004577
IFA	FA 22:1	0.0111035	0.0065041
IFA	FA 22:2	0.0005387	0.0002785
IFA	FA 22:6	0.0015892	0.0007912
IFA	FA 24:0	0.0295301	0.0118436
IFA	FA 24:1	0.0028836	0.0017223
IFA	FA 26:0	0.0102839	0.0050825
IFA	FA 28:0	0.0034348	0.0017696
IFA	GlcCer 40:1 d.1	0.0026785	0.0007077
IFA	GlcCer 41:1 d	0.0014515	0.0003318
IFA	GlcCer 42:1 d.1	0.0052196	0.0014145
IFA	GlcCer 14:1 d	0.0002688	0.0000843
IFA	LPC 18:1	0.0074064	0.0067438
IFA	LPE 20:4	0.0017508	0.0007614
IFA	PC 34:1 ox	0.1782963	0.120497
IFA	PC 36:1 ox	0.0894071	0.0639421
IFA	PC 36:3 ox	0.0760728	0.0494009
IFA	PC 36:4 ox	0.0108022	0.0096028
IFA	PC 38:3 ox	0.0342822	0.0228373
IFA	PC 38:5 ox	0.0875128	0.0548806
IFA	PC 32:2	0.041125	0.0218242
IFA	PC 33:1.1	0.0120778	0.0041261
IFA	PC 34:3.1	0.0423983	0.0143412
IFA	PC 34:4	0.0123839	0.0064009

IFA	PC 35:1.1	0.0183162	0.0046394
IFA	PC 35:2.1	0.0471128	0.0104684
IFA	PC 35:4.1	0.0065557	0.0013707
IFA	PC 36:3.3	0.7124727	0.1437203
IFA	PC 36:5.2	0.0053601	0.002698
IFA	PC 37:2	0.0109624	0.002906
IFA	PC 38:3.1	0.3452416	0.0877722
IFA	PC 40:4.1	0.0237669	0.0086024
IFA	PC 40:5.2	0.0207383	0.00731
IFA	PC 40:6.2	0.0052069	0.0020789
IFA	PC 40:8.1	0.0144949	0.003709
IFA	PC 32:0 p.1	0.0224867	0.0059452
IFA	PC 34:0 p	0.049042	0.0107566
IFA	PC 34:1 p.1	0.0301399	0.0081112
IFA	PC 34:2 p.1	0.083143	0.0155139
IFA	PC 36:1 p.1	0.013587	0.0035097
IFA	PC 36:3 p.1	0.2078383	0.0669219
IFA	PC 36:4 p.1	0.1552032	0.0535158
IFA	PC 38:4 p.2	0.1615937	0.0640932
IFA	PC 38:5 p.1	0.0370738	0.0165683
IFA	PC 40:3 p.1	0.0112956	0.0026442
IFA	PC 40:4 p.1	0.0171801	0.0050821
IFA	PC 42:4 p	0.0142124	0.0041205
IFA	PC 42:5 p.1	0.0053381	0.0018361
IFA	PC 44:4 p	0.0196414	0.0054217
IFA	PE 34:1	0.0195649	0.0193494
IFA	PE 36:2	0.0857042	0.0536013
IFA	PE 36:3	0.0296203	0.0215375
IFA	PE 36:4.1	0.0588403	0.0583922
IFA	PE 38:2	0.1145517	0.0500675
IFA	PE 38:4	0.109448	0.0790307
IFA	PE 38:6.1	0.1090591	0.0714314
IFA	PE 40:6	0.0578348	0.0233688
IFA	PE 34:1 p.1	0.0336497	0.015536
IFA	PE 34:2 p	0.0649856	0.0326308
IFA	PE 36:1 p	0.0245823	0.0138358
IFA	PE 36:2 p.1	0.088318	0.0536387
IFA	PE 36:4 p.1	0.320295	0.1646583
IFA	PE 36:5 p	0.0511178	0.03858
IFA	PE 38:3 p	0.0174866	0.0110981
IFA	PE 38:4 p.1	0.3041991	0.1729397
IFA	PE 38:5 p.1	0.1340207	0.0639391
IFA	PE 38:6 p	0.201743	0.0900529

IFA	PE 40:4 p	0.0417752	0.0204789
IFA	PE 40:5 p.1	0.0236518	0.0112022
IFA	PE 40:6 p	0.1680759	0.06853
IFA	PE 40:7 p	0.0818823	0.0371542
IFA	PE 40:6.1	0.070704	0.0521237
IFA	SM 32:1 d	0.6040555	0.2432702
IFA	SM 33:1 d	0.1027727	0.0272922
IFA	SM 34:1 d	0.1409922	0.0206633
IFA	SM 39:1 d	0.1490923	0.0357333
IFA	SM 40:2 d.1	0.597116	0.1536945
IFA	SM 40:3 d	0.039853	0.0158486
IFA	SM 41:1 d	0.5657862	0.1712711
IFA	SM 41:2 d	0.2437669	0.0814548
IFA	SM 42:1 d.1	1.8715929	0.5188128
IFA	SM 43:2 d	0.0258866	0.0088591
SQ-LNS	CE 16:1	0.1172686	0.0375424
SQ-LNS	CE 18:1	2.5704347	0.8078552
SQ-LNS	CE 18:2	16.5461683	3.7542437
SQ-LNS	CE 18:3	0.1422087	0.0491212
SQ-LNS	CE 20:3	0.2404385	0.0767924
SQ-LNS	CE 20:4	1.9353538	0.8541507
SQ-LNS	CE 20:5	0.4098558	0.2457458
SQ-LNS	CE 22:6	0.6411996	0.2085378
SQ-LNS	Ceramide 42:2 d	0.0084946	0.0293267
SQ-LNS	Cholesterol	7.9439618	1.0939401
SQ-LNS	DG 32:1	0.0306967	0.0196531
SQ-LNS	DG 34:1	0.1886315	0.2178358
SQ-LNS	DG 34:2	0.262915	0.8738713
SQ-LNS	DG 34:3	0.0078732	0.0051651
SQ-LNS	DG 36:1	0.0355209	0.0699222
SQ-LNS	DG 36:2	0.240809	0.5201445
SQ-LNS	DG 36:3	0.2203406	0.1780222
SQ-LNS	DG 36:4 A	0.0445387	0.045145
SQ-LNS	DG 38:5	0.0552547	0.1400318
SQ-LNS	DG 38:6	0.0297175	0.0429936
SQ-LNS	GlcCer 40:1 d	0.006362	0.0015482
SQ-LNS	GlcCer 42:1 d	0.010395	0.002391
SQ-LNS	GlcCer 42:2 d	0.0107109	0.0028466
SQ-LNS	Gal-Gal-Cer 42:2 d	0.0009497	0.0004893
SQ-LNS	LPC 20:0	0.0013124	0.0006989
SQ-LNS	LPC 20:1	0.0010376	0.0006871
SQ-LNS	LPC 22:4	0.0013313	0.0013149
SQ-LNS	LPC 22:6	0.0030319	0.0034239

SQ-LNS	LPC 16:0 o	0.0037945	0.0034384
SQ-LNS	LPC 16:0 p	0.0027348	0.0018153
SQ-LNS	LPC 16:0	0.0477863	0.0278614
SQ-LNS	LPC 18:0	0.0866232	0.0450481
SQ-LNS	LPC 18:2	0.0072848	0.0138528
SQ-LNS	LPC 20:3	0.0009618	0.0013843
SQ-LNS	LPC 20:4	0.0043289	0.0044051
SQ-LNS	LPC 20:5	0.0005992	0.0005718
SQ-LNS	PC 25:0	0.0048714	0.0038456
SQ-LNS	PC 37:3	0.0068339	0.0022003
SQ-LNS	PC 39:6	0.0192601	0.0095333
SQ-LNS	PC 42:5	0.0038281	0.0017599
SQ-LNS	PC 42:6	0.0018966	0.0011516
SQ-LNS	PC 32:0 p	0.0336667	0.0101927
SQ-LNS	PC 36:3 p	0.265237	0.0733309
SQ-LNS	PC 38:2 p	0.0071739	0.0020064
SQ-LNS	PC 42:3 p	0.0069965	0.0023865
SQ-LNS	PC 34:1	0.0623357	0.0193641
SQ-LNS	PC 36:3	0.0743385	0.0155648
SQ-LNS	PC 36:5	0.6194141	0.143297
SQ-LNS	PC 38:4	0.0351583	0.0103654
SQ-LNS	PC 40:6	0.0265605	0.0096341
SQ-LNS	PC 28:0	0.005046	0.0034233
SQ-LNS	PC 30:0	0.1312689	0.0783405
SQ-LNS	PC 31:0	0.0033269	0.001386
SQ-LNS	PC 32:0	0.1653747	0.0671799
SQ-LNS	PC 32:1	0.1588391	0.0835923
SQ-LNS	PC 33:0	0.0044926	0.002161
SQ-LNS	PC 33:1	0.0230748	0.0095425
SQ-LNS	PC 33:2	0.023875	0.0082652
SQ-LNS	PC 34:0	0.0308811	0.0055635
SQ-LNS	PC 34:1.1	5.4348017	1.9297814
SQ-LNS	PC 34:2	12.4044609	2.7498819
SQ-LNS	PC 34:3	0.0237397	0.018558
SQ-LNS	PC 35:1	0.0251091	0.0081639
SQ-LNS	PC 35:2	0.0614211	0.0262102
SQ-LNS	PC 35:3	0.0132715	0.0052922
SQ-LNS	PC 35:4	0.0069946	0.0019665
SQ-LNS	PC 36:1	0.5897235	0.2094777
SQ-LNS	PC 36:2	6.0514834	1.5973228
SQ-LNS	PC 36:3.1	0.9733252	0.3439876
SQ-LNS	PC 36:3.2	1.0874944	0.3132306
SQ-LNS	PC 36:4	0.2041814	0.0973363

SQ-LNS	PC 36:4.1	0.0721607	0.0339318
SQ-LNS	PC 36:5.1	0.0393919	0.1296014
SQ-LNS	PC 36:6	0.0121756	0.006808
SQ-LNS	PC 37:4	0.0186188	0.0103226
SQ-LNS	PC 37:6	0.0063777	0.0019863
SQ-LNS	PC 38:2	0.0397587	0.0093036
SQ-LNS	PC 38:3	0.4119036	0.1217888
SQ-LNS	PC 38:4.1	0.0810662	0.0285069
SQ-LNS	PC 38:5	0.6215453	0.189588
SQ-LNS	PC 38:5.1	0.2952537	0.1508099
SQ-LNS	PC 38:6	7.1127158	1.9166711
SQ-LNS	PC 38:7	0.0066044	0.0034111
SQ-LNS	PC 40:4	0.0248568	0.0077324
SQ-LNS	PC 40:5	0.2616236	0.0900439
SQ-LNS	PC 40:5.1	0.0323922	0.0134681
SQ-LNS	PC 40:6.1	1.2156765	0.4251956
SQ-LNS	PC 40:7	0.0126845	0.0275439
SQ-LNS	PC 40:8	0.0134552	0.0050849
SQ-LNS	PC 32:0 o	0.0281432	0.0101377
SQ-LNS	PC 34:0 o	0.0028988	0.00106
SQ-LNS	PC 32:1 p	0.006231	0.0019771
SQ-LNS	PC 34:1 p	0.0757199	0.0247489
SQ-LNS	PC 34:2 p	0.1119818	0.0288562
SQ-LNS	PC 36:1 p	0.0040475	0.0011834
SQ-LNS	PC 36:2 p	0.0140285	0.0032869
SQ-LNS	PC 36:4 p	0.3457668	0.1184345
SQ-LNS	PC 38:3 p	0.079174	0.0206566
SQ-LNS	PC 38:4 p	0.2729425	0.0811178
SQ-LNS	PC 38:4 p.1	0.0490453	0.0146622
SQ-LNS	PC 38:5 p	0.1369679	0.0435463
SQ-LNS	PC 40:3 p	0.0113999	0.0034688
SQ-LNS	PC 40:4 p	0.0208587	0.0056971
SQ-LNS	PC 40:5 p	0.0376691	0.0103214
SQ-LNS	PC 40:6 p	0.0960779	0.0340754
SQ-LNS	PC 40:6 p.1	0.0360321	0.0130526
SQ-LNS	PC 42:5 p	0.0056704	0.0025689
SQ-LNS	PE 34:3	0.1148354	0.0742051
SQ-LNS	PE 38:7	0.4085992	0.2686413
SQ-LNS	PE 34:2	0.0834202	0.0403411
SQ-LNS	PE 36:1	0.0279161	0.0130278
SQ-LNS	PE 36:4	0.070651	0.0298363
SQ-LNS	PE 38:6	0.1940924	0.0876449
SQ-LNS	PE 34:1 p	0.0446616	0.0327414

SQ-LNS	PE 36:2 p	0.1029268	0.0592955
SQ-LNS	PE 36:4 p	0.4953766	0.3926091
SQ-LNS	PE 38:4 p	0.4232692	0.2942923
SQ-LNS	PE 38:5 p	0.2318585	0.1721598
SQ-LNS	PE 40:5 p	0.0535427	0.030376
SQ-LNS	SM 37:1 d	0.0082721	0.0021362
SQ-LNS	SM 38:0 d	0.0084384	0.0034748
SQ-LNS	SM 39:2 d	0.0058114	0.0014984
SQ-LNS	SM 42:0 d	0.0040643	0.0016704
SQ-LNS	SM 44:2 d	0.0040925	0.001768
SQ-LNS	SM 42:1 d	0.0274527	0.0114634
SQ-LNS	SM 30:1 d	0.0067356	0.0034033
SQ-LNS	SM 32:0 d	0.0035501	0.0018594
SQ-LNS	SM 32:2 d	0.0096374	0.0038489
SQ-LNS	SM 34:0 d	0.0583558	0.0178946
SQ-LNS	SM 34:2 d	0.1582544	0.0336282
SQ-LNS	SM 36:0 d	0.0272507	0.012595
SQ-LNS	SM 36:1 d	0.2285555	0.0625031
SQ-LNS	SM 36:2 d	0.2886074	0.075667
SQ-LNS	SM 36:3 d	0.0036113	0.0013661
SQ-LNS	SM 38:1 d	0.1346627	0.0294519
SQ-LNS	SM 38:2 d	0.0719533	0.0161779
SQ-LNS	SM 40:0 d	0.0120294	0.0052342
SQ-LNS	SM 40:1 d	0.2754813	0.0549353
SQ-LNS	SM 40:2 d	0.1443573	0.0278784
SQ-LNS	SM 42:2 d	0.6287308	0.1752583
SQ-LNS	SM 42:3 d	0.3302789	0.071043
SQ-LNS	SM 43:1 d	0.001799	0.0011482
SQ-LNS	TG 52:4	0.0332221	0.0129615
SQ-LNS	TG 40:0	0.0078556	0.0046118
SQ-LNS	TG 42:0	0.0107287	0.0070818
SQ-LNS	TG 42:1	0.0068933	0.0054528
SQ-LNS	TG 42:2	0.0050585	0.0035522
SQ-LNS	TG 42:3	0.0047871	0.0050918
SQ-LNS	TG 44:0	0.0261402	0.0150106
SQ-LNS	TG 44:1	0.0264604	0.0221281
SQ-LNS	TG 46:0	0.105516	0.0543367
SQ-LNS	TG 46:1	0.0867889	0.0616148
SQ-LNS	TG 46:2	0.0379196	0.0273824
SQ-LNS	TG 46:3	0.003257	0.0021997
SQ-LNS	TG 48:1	0.5626962	0.2925713
SQ-LNS	TG 48:2	0.1438373	0.0804847
SQ-LNS	TG 48:3	0.0460452	0.0327937

SQ-LNS	TG 48:4	0.0083208	0.0071226
SQ-LNS	TG 49:1	0.0041954	0.0018325
SQ-LNS	TG 49:2	0.0169838	0.0075757
SQ-LNS	TG 49:3	0.0033585	0.0023448
SQ-LNS	TG 50:0	0.058592	0.0267954
SQ-LNS	TG 50:1	0.9162823	0.8320556
SQ-LNS	TG 50:2	1.1478158	0.4073598
SQ-LNS	TG 50:3	0.2417879	0.1049906
SQ-LNS	TG 50:4	0.042297	0.0238009
SQ-LNS	TG 50:5	0.0044362	0.0023304
SQ-LNS	TG 51:1	0.019202	0.0079921
SQ-LNS	TG 51:2	0.0341554	0.0138308
SQ-LNS	TG 51:3	0.0174202	0.0062931
SQ-LNS	TG 51:4	0.0037143	0.0015339
SQ-LNS	TG 52:1	0.2348302	0.1103584
SQ-LNS	TG 52:2	2.248459	1.4384472
SQ-LNS	TG 52:3	2.5911007	0.9890893
SQ-LNS	TG 52:4.1	0.6914702	0.3350214
SQ-LNS	TG 52:5	0.0359948	0.0194739
SQ-LNS	TG 52:6	0.006045	0.0033895
SQ-LNS	TG 53:1	0.007795	0.0034793
SQ-LNS	TG 53:2	0.0218266	0.0112691
SQ-LNS	TG 53:3	0.0212971	0.0085751
SQ-LNS	TG 53:4	0.010304	0.0034295
SQ-LNS	TG 54:1	0.0315867	0.0173837
SQ-LNS	TG 54:2	0.1784553	0.0890126
SQ-LNS	TG 54:3	0.7259255	0.3178531
SQ-LNS	TG 54:4	0.5246409	0.26219
SQ-LNS	TG 54:5	0.161007	0.0588594
SQ-LNS	TG 54:6	0.0611312	0.0240471
SQ-LNS	TG 56:3	0.0110907	0.0048798
SQ-LNS	TG 56:4	0.0138708	0.0091145
SQ-LNS	TG 56:5	0.0413495	0.0199761
SQ-LNS	TG 56:6	0.1104069	0.0390809
SQ-LNS	TG 56:7	0.193695	0.1161735
SQ-LNS	TG 56:8	0.0017132	0.0010113
SQ-LNS	TG 57:1	0.0065229	0.0060171
SQ-LNS	TG 58:1	0.0045234	0.003258
SQ-LNS	TG 58:3	0.0031279	0.0017908
SQ-LNS	TG 58:6	0.0055387	0.0023034
SQ-LNS	TG 58:8	0.0045036	0.0024864
SQ-LNS	TG 58:9	0.0317853	0.0172436
SQ-LNS	Ceramide 32:1 d	0.0013969	0.0068059

SQ-LNS	Ceramide 34:0 d	0.0008794	0.0019474
SQ-LNS	Ceramide 34:1 d	0.0121097	0.0578162
SQ-LNS	Ceramide 34:2 d	0.0047711	0.0283305
SQ-LNS	Ceramide 36:1 d	0.0028992	0.0124878
SQ-LNS	Ceramide 38:1 d	0.0025634	0.0117642
SQ-LNS	Ceramide 39:1 d	0.0007598	0.0028978
SQ-LNS	Ceramide 40:0 d	0.0012374	0.0009647
SQ-LNS	Ceramide 40:1 d	0.0068511	0.0217862
SQ-LNS	Ceramide 40:2 d	0.0034911	0.0189749
SQ-LNS	Ceramide 41:1 d	0.0034823	0.0065247
SQ-LNS	Ceramide 42:0 d	0.0017815	0.0009398
SQ-LNS	Ceramide 42:1 d	0.0187138	0.0217754
SQ-LNS	Ceramide 44:1 d	0.000287	0.0001911
SQ-LNS	FA 12:0	0.0052904	0.0049265
SQ-LNS	FA 13:0	0.0012885	0.0021053
SQ-LNS	FA 14:0	0.0341244	0.0584196
SQ-LNS	FA 14:1	0.004127	0.0175151
SQ-LNS	FA 15:0	0.0337536	0.0961399
SQ-LNS	FA 15:1	0.0064931	0.0262332
SQ-LNS	FA 16:0	1.6806879	1.4411496
SQ-LNS	FA 16:1	0.0668977	0.2472031
SQ-LNS	FA 17:0	0.0328986	0.054919
SQ-LNS	FA 18:1	0.1799265	0.1857238
SQ-LNS	FA 18:2	0.0358925	0.0417978
SQ-LNS	FA 18:3	0.0015126	0.0023479
SQ-LNS	FA 20:0	0.0519653	0.0491859
SQ-LNS	FA 20:1	0.0076104	0.0108292
SQ-LNS	FA 20:2	0.0033988	0.0105294
SQ-LNS	FA 20:3	0.0008934	0.002721
SQ-LNS	FA 20:3.1	0.0008502	0.0007836
SQ-LNS	FA 20:4	0.0026703	0.0021525
SQ-LNS	FA 20:5	0.0007859	0.0006339
SQ-LNS	FA 22:1	0.0102528	0.006256
SQ-LNS	FA 22:2	0.0010511	0.0025574
SQ-LNS	FA 22:6	0.0018178	0.00168
SQ-LNS	FA 24:0	0.0445648	0.0795674
SQ-LNS	FA 24:1	0.0031482	0.0030622
SQ-LNS	FA 26:0	0.0132946	0.0170698
SQ-LNS	FA 28:0	0.0038908	0.0036526
SQ-LNS	GlcCer 40:1 d.1	0.0029535	0.000727
SQ-LNS	GlcCer 41:1 d	0.0015315	0.0003807
SQ-LNS	GlcCer 42:1 d.1	0.0056147	0.001429
SQ-LNS	GlcCer 14:1 d	0.0002745	0.0000974

SQ-LNS	LPC 18:1	0.0064346	0.0047371
SQ-LNS	LPE 20:4	0.0015897	0.0007904
SQ-LNS	PC 34:1 ox	0.1633026	0.1117518
SQ-LNS	PC 36:1 ox	0.0869455	0.0667646
SQ-LNS	PC 36:3 ox	0.0680817	0.0460763
SQ-LNS	PC 36:4 ox	0.0089308	0.0058718
SQ-LNS	PC 38:3 ox	0.0333052	0.0245745
SQ-LNS	PC 38:5 ox	0.0788452	0.0466738
SQ-LNS	PC 32:2	0.035841	0.0166356
SQ-LNS	PC 33:1.1	0.0103018	0.0042478
SQ-LNS	PC 34:3.1	0.0384133	0.014787
SQ-LNS	PC 34:4	0.010816	0.0055391
SQ-LNS	PC 35:1.1	0.0166963	0.0053629
SQ-LNS	PC 35:2.1	0.0438978	0.0108403
SQ-LNS	PC 35:4.1	0.0057735	0.0015945
SQ-LNS	PC 36:3.3	0.6666642	0.1950993
SQ-LNS	PC 36:5.2	0.0047325	0.0022712
SQ-LNS	PC 37:2	0.0107976	0.0030357
SQ-LNS	PC 38:3.1	0.3513382	0.1000436
SQ-LNS	PC 40:4.1	0.0227492	0.0074492
SQ-LNS	PC 40:5.2	0.0191121	0.0081442
SQ-LNS	PC 40:6.2	0.0046972	0.0017273
SQ-LNS	PC 40:8.1	0.0132413	0.004149
SQ-LNS	PC 32:0 p.1	0.0224336	0.0059068
SQ-LNS	PC 34:0 p	0.0486178	0.0154074
SQ-LNS	PC 34:1 p.1	0.0268755	0.0089101
SQ-LNS	PC 34:2 p.1	0.0758884	0.0183447
SQ-LNS	PC 36:1 p.1	0.012438	0.0038519
SQ-LNS	PC 36:3 p.1	0.1951722	0.0527709
SQ-LNS	PC 36:4 p.1	0.1466776	0.0458757
SQ-LNS	PC 38:4 p.2	0.1466107	0.0413566
SQ-LNS	PC 38:5 p.1	0.0350587	0.0101234
SQ-LNS	PC 40:3 p.1	0.0106764	0.0032772
SQ-LNS	PC 40:4 p.1	0.0161831	0.0043761
SQ-LNS	PC 42:4 p	0.012957	0.0043903
SQ-LNS	PC 42:5 p.1	0.004857	0.0017338
SQ-LNS	PC 44:4 p	0.0184854	0.0062327
SQ-LNS	PE 34:1	0.0147663	0.0076946
SQ-LNS	PE 36:2	0.082601	0.041408
SQ-LNS	PE 36:3	0.0280081	0.0161827
SQ-LNS	PE 36:4.1	0.046685	0.0303738
SQ-LNS	PE 38:2	0.1239872	0.0771388
SQ-LNS	PE 38:4	0.1037742	0.0610657

SQ-LNS	PE 38:6.1	0.0942571	0.044743
SQ-LNS	PE 40:6	0.0628568	0.0409313
SQ-LNS	PE 34:1 p.1	0.0455344	0.06819
SQ-LNS	PE 34:2 p	0.0655866	0.0452342
SQ-LNS	PE 36:1 p	0.0285322	0.0307308
SQ-LNS	PE 36:2 p.1	0.0837786	0.0496689
SQ-LNS	PE 36:4 p.1	0.4020645	0.3676741
SQ-LNS	PE 36:5 p	0.0649994	0.0530424
SQ-LNS	PE 38:3 p	0.0182673	0.0145774
SQ-LNS	PE 38:4 p.1	0.35287	0.2757311
SQ-LNS	PE 38:5 p.1	0.1680656	0.136812
SQ-LNS	PE 38:6 p	0.2507388	0.1802873
SQ-LNS	PE 40:4 p	0.0432454	0.0275944
SQ-LNS	PE 40:5 p.1	0.0256717	0.0165422
SQ-LNS	PE 40:6 p	0.2018203	0.1421401
SQ-LNS	PE 40:7 p	0.103705	0.0775974
SQ-LNS	PE 40:6.1	0.0614635	0.0369444
SQ-LNS	SM 32:1 d	0.6388281	0.3067823
SQ-LNS	SM 33:1 d	0.1008706	0.0242546
SQ-LNS	SM 34:1 d	0.139326	0.0294711
SQ-LNS	SM 39:1 d	0.1617093	0.0452437
SQ-LNS	SM 40:2 d.1	0.6006079	0.2135754
SQ-LNS	SM 40:3 d	0.0402978	0.012407
SQ-LNS	SM 41:1 d	0.5686648	0.15228
SQ-LNS	SM 41:2 d	0.2463125	0.0669829
SQ-LNS	SM 42:1 d.1	1.8647426	0.4814959
SQ-LNS	SM 43:2 d	0.0267404	0.011101

Table S2. 33 HDL Associated Proteins.			
Treatment	Protein	Mean Abundance	SD
IFA	APOC1	10811.85928	4721.299434
IFA	APOA1	1434574.094	537468.0296
IFA	FETUA	33573.28762	17359.22421
IFA	PLTP	5623.374983	4457.277207
IFA	APOD	329722.0244	124745.4338
IFA	SAA4	32818.62972	11662.22981
IFA	APOL1	8561.503842	8570.663364
IFA	AACT	5297.747485	3508.021458
IFA	APOE	61137.52336	42382.86114
IFA	A1AT	61361.18929	22378.9738
IFA	APOA2	194283.795	89854.10462
IFA	C1S	2074.737315	1108.66985
IFA	APOC2	242650.1624	190106.9337
IFA	APOA4	11295.49729	9835.211986
IFA	LCAT	2754.608693	1701.719108
IFA	A1BG	76818.44814	51910.24348
IFA	APOC4	975.8648138	949.1820964
IFA	APOM	853981.3669	288462.2957
IFA	C3	398.4395162	219.1307649
IFA	SAA2	14229.66271	9066.199282
IFA	SAA1	16262.24619	30428.87241
IFA	APOA	7.557435965	6.499382815
IFA	HPX	7562.57927	5447.714586
IFA	APOC3	46241.45447	57844.54375
IFA	APOF	1561.646202	1008.007114
IFA	HCF2	2914.654609	1608.458057
IFA	KNG1	7765.542371	6369.46123
IFA	APOH	1854.631842	1464.540365
IFA	PON1	16.96993885	21.56828186
IFA	CLUS	2545.537839	1228.904949
IFA	APOB	4325.351691	2240.120226
IFA	PON3	643.8255329	644.2029852
IFA	APOA5	9.084599445	6.937561639
SQ-LNS	APOC1	24343.81273	74660.13338
SQ-LNS	APOA1	1527916.768	468350.9347
SQ-LNS	FETUA	30807.90691	30708.42266
SQ-LNS	PLTP	5928.588681	3818.181819
SQ-LNS	APOD	347311.1365	137578.4109
SQ-LNS	SAA4	36651.03133	20392.23984
SQ-LNS	APOL1	7836.096267	6491.720431

SQ-LNS	AACT	4511.620293	4065.214564
SQ-LNS	APOE	73933.4948	62933.875
SQ-LNS	A1AT	63538.09406	38373.98613
SQ-LNS	APOA2	210157.6142	105517.1161
SQ-LNS	C1S	1755.865029	1243.31605
SQ-LNS	APOC2	252766.0797	165642.9704
SQ-LNS	APOA4	8757.442428	6441.141791
SQ-LNS	LCAT	3013.289631	1983.950556
SQ-LNS	A1BG	66299.90019	75130.04769
SQ-LNS	APOC4	1290.926044	1192.282178
SQ-LNS	APOM	804693.8012	281472.0627
SQ-LNS	C3	471.9884806	410.1395085
SQ-LNS	SAA2	16249.56888	9897.608383
SQ-LNS	SAA1	36468.54807	159310.3319
SQ-LNS	APOA	7.498998449	9.154410747
SQ-LNS	HEMO	7573.108655	10014.01744
SQ-LNS	APOC3	46608.03706	37034.22342
SQ-LNS	APOF	1848.63341	1251.221326
SQ-LNS	HECF	2476.216139	1802.009654
SQ-LNS	KNG1	7245.733778	9483.517209
SQ-LNS	APOH	2032.34937	2009.99746
SQ-LNS	PON1	38.54713922	131.8124813
SQ-LNS	CLUS	2777.09503	1152.797746
SQ-LNS	APOB	4911.270736	2882.565501
SQ-LNS	PON3	751.040393	652.1189335
SQ-LNS	APOA5	11.42376376	11.22191583

**Table S3**. Glycopeptides analysis from 21 HDL associated proteins.			
Treatment	Glycopeptides (n = 163)	Mean Abundance	SD
IFA	APOB_3224_54020_z3	0.0394947	0.0172598
IFA	APOB_3411_5401	0.0128901	0.0227725
IFA	AACT_271_6512	0.0078521	0.0076865
IFA	AACT_271_7603	0.031695	0.0255303
IFA	AACT_271_7602	0.0482093	0.0290721
IFA	AACT_271_6502	0.0020941	0.0022993
IFA	AACT_106_7603	0.0021432	0.0015894
IFA	APOB_185_52000_z3	0.8218095	1.5675627
IFA	APOB_983_5401	0.0252867	0.0106358
IFA	AACT_106_7604	0.0005456	0.0006506
IFA	A1BG_179_5421/5402	0.0178749	0.0039714
IFA	APOB_983_54020_z4	0.0412918	0.0144838
IFA	AACT_127_5401	0.0018222	0.0013482
IFA	AACT_127_8500	0.0454916	0.052064
IFA	A1AT_271_5412	0.0005245	0.0004244
IFA	A1AT_271_5402	0.0527909	0.0206704
IFA	A1AT_271_55110_z3	0.0002886	0.0002232
IFA	A1AT_271_5401	0.0005451	0.0003676
IFA	A1AT_271_6503	0.0002403	0.0002031
IFA	APOA2_35_11020_z3	0.0010285	0.0008335
IFA	APOA2_35_11010_z2	0.0000227	0.0000456
IFA	A1AT_271MC_5412	0.005606	0.00441
IFA	A1AT_107_5401	0.0174233	0.0101624
IFA	A1AT_107_5411	0.0356425	0.009788
IFA	A1AT_271MC_5402	0.1437275	0.0444519
IFA	A1AT_107_6512	0.0103113	0.0077278
IFA	A1AT_107_6502/6521	0.0265805	0.0107613
IFA	A1AT_107_5402	0.0606979	0.0154517
IFA	A1AT_107_5412	0.0051522	0.0021896
IFA	A1AT_107_6513	0.0084881	0.0058065
IFA	A1AT_107_6503	0.0256588	0.0102744
IFA	A1AT_107_6501/6520	0.0020917	0.0050324
IFA	APOA2_95_11010_z2	0.0003368	0.0001962
IFA	APOA2_88_11010_z2	0.0002829	0.0001727
IFA	A1AT_70_5412	0.0008311	0.0003813
IFA	A1AT_70_5402	0.0503268	0.0193743
IFA	APOD_65_65210_z3	0.0000209	0.0000178
IFA	APOD_65_65030_z3	0.000045	0.000044
IFA	APOD_98_54100_z3	0.0013688	0.0015123
IFA	APOD_98_54000_z3	0.0001259	0.0000645

IFA	APOD_65_65020_z3	0.0000239	0.0000288
IFA	APOD_65_65010_z3	0.0000257	0.000018
IFA	APOD_65_65020_z3_deamidated	0.0001719	0.0001575
IFA	APOD_98_65010_z3	0.0000192	0.0000233
IFA	APOD_98_65110_z3	0.000041	0.0000257
IFA	APOD_65_54010_z3	0.0000572	0.0000369
IFA	APOD_98_54110_z3	0.000161	0.0000881
IFA	APOD_98_54020_z3	0.0000193	0.0000221
IFA	APOD_98_54010_z3	0.0000907	0.0000686
IFA	APOD_65_54020_z3	0.000032	0.0000238
IFA	APOD_98_65130_z3	0.0000244	0.0000252
IFA	APOD_98_54120_z3	0.0000332	0.00003
IFA	APOD_65_76020_z3	0.0000297	0.000028
IFA	APOD_98_5402	0.0004665	0.0002325
IFA	APOD_98_65320_z3	0.0000072	0.0000077
IFA	APOD_65_54200_z3	0.0000498	0.0000387
IFA	APOD_65_76210_z3	0.0000263	0.0000226
IFA	APOD_65_65030_z3_deamidated	0.0000185	0.0000137
IFA	APOD_98_6520	0.0002007	0.0001675
IFA	APOD_98_6510	0.0005036	0.0004456
IFA	APOD_98_9800	0.0003908	0.0006734
IFA	APOD_98_6530	0.0006519	0.0005456
IFA	APOD_98_7600	0.0002658	0.0002406
IFA	APOC3_94AMC_12020_z3	0.0256366	0.0418916
IFA	APOC3_94AMC_22100_z3	0.0008421	0.0013659
IFA	APOC3_94_1210	0.0011263	0.0014675
IFA	APOC3_94_0310	0.0007523	0.0008252
IFA	APOC3_94_0300	0.1284821	0.1720296
IFA	APOC3_94AMC_11000_z3	0.0048801	0.0022002
IFA	APOC3_94MC_11010_z3	0.0273929	0.0125607
IFA	APOC3_94A_11010_z2	0.0010305	0.0008245
IFA	APOC3_94MC_1102	0.1616978	0.031748
IFA	APOC3_94MC_11020_z3	0.0112174	0.0057145
IFA	APOC3_94_1101	0.1479717	0.0421585
IFA	APOC3_94A_11000_z2	0.0006624	0.0005272
IFA	APOC3_94Aoff_1101	0.0080437	0.0048694
IFA	APOC3_94_2110	0.0009794	0.0012795
IFA	APOC3_94Aoff_1102	0.0056151	0.0045721
IFA	APOC3_94_1102	0.1055842	0.023329
IFA	APOC3_94A_11020_z2	0.0002285	0.000188
IFA	APOC3_94_1111	0.0056157	0.0138182
IFA	APOC3_94_1300	0.0030746	0.0043121
IFA	APOC3_94_1202	0.0057645	0.0081593

IFA	CLUS_291_5400	0.0573338	0.0744249
IFA	CLUS_291_6503	0.018689	0.0390782
IFA	CLUS_291_54020_z3	0.1482094	0.0418989
IFA	CLUS_291_5421/5402	0.1232955	0.0391082
IFA	CLUS_291_5420/5401	0.0408822	0.0241741
IFA	CLUS_86_6503	0.0013877	0.0007987
IFA	APOF_269_11010_z2	0.0808883	0.0227037
IFA	APOF_273/274_11010_z3	2.251669	0.4186461
IFA	APOF_273/274_11020_z3	0.9569323	0.1903533
IFA	APOE_307/308_11010_z3	0.038893	0.0090965
IFA	APOE_307/308_11010_z2	0.0010153	0.0003066
IFA	CLUS_374_6520/6501	0.1244688	0.0534979
IFA	CLUS_374_5402/5421	0.106659	0.0333114
IFA	APOE_215_11010_z2	0.0062528	0.0067569
IFA	CLUS_374_5420/5401	1.7521068	1.1364774
IFA	APOE_215_11020_z2	0.001846	0.0016198
IFA	CLUS_374_6512	0.1395672	0.0940911
IFA	CO3_85_6200	0.5173679	0.2220976
IFA	CO3_85_5200	0.4647495	0.218606
IFA	CO3_85_7200	0.2139657	0.1496251
IFA	APOM_135_43010_z3	0.0006191	0.0002381
IFA	APOM_135_54010_z3	0.00039	0.0000915
IFA	APOM_135_53010_z3	0.0001145	0.0000548
IFA	CO3_85_6301	1.0192401	0.7802088
IFA	APOM_135_54020_z3	0.000154	0.0000673
IFA	APOM_135_44010_z3	0.0000445	0.0000384
IFA	APOM_135_55110_z3	0.0000304	0.0000166
IFA	C1S_174_5402	0.0083218	0.0048297
IFA	C1S_174_5401	0.0092951	0.0068835
IFA	APOM_135_5421	0.0001529	0.0001102
IFA	APOM_135_8500	0.0000314	0.0000162
IFA	HEMO_240/246_5402	0.0015473	0.0016394
IFA	HEMO_187_6503	0.0186528	0.005605
IFA	HEMO_187_5402/5421	0.0904443	0.012613
IFA	HEMO_187_5412/5431	0.0045839	0.0029389
IFA	FETUA_346_1101	0.644887	0.1071992
IFA	FETUA_346_2200	0.0021247	0.0012919
IFA	FETUA_156_6411	0.0020513	0.0016073
IFA	FETUA_156_5400	0.0080567	0.0056716
IFA	FETUA_156_5401	0.0370009	0.0111281
IFA	HEP2_49_5402	0.3926462	0.2115345
IFA	FETUA_156_6410	0.0150072	0.0089408
IFA	FETUA_156_6502	0.0262995	0.0097264

IFA	FETUA_156_5402/5421	0.4521056	0.0788971
IFA	FETUA_156_6503	0.0258202	0.0103454
IFA	FETUA_176_6501	0.0272377	0.0176713
IFA	HEP2_49_5412	0.019682	0.0158072
IFA	FETUA_156_6513	0.003414	0.0030239
IFA	HEMO_453_5420/5401	0.148944	0.0538416
IFA	FETUA_176_5401	0.0883577	0.033112
IFA	FETUA_176_6512	0.0063118	0.0036247
IFA	FETUA_176_6502	0.0114561	0.0050218
IFA	FETUA_176_6513	0.004584	0.0027493
IFA	FETUA_176_7600	0.0415096	0.0111661
IFA	FETUA_176_5412/5431	0.0213714	0.0057454
IFA	FETUA_176_5402/5421	0.1178337	0.0147793
IFA	FETUA_176_6503	0.0083317	0.00254
IFA	HEMO_453_5402/5421	0.1625453	0.0366154
IFA	LCAT_108_65030_z3	0.0146358	0.0045639
IFA	KNG1_205_6503	0.006725	0.0035589
IFA	KNG1_205_5402/5421	0.0035403	0.0019222
IFA	KNG1_205_6512	0.0069067	0.0081522
IFA	PON3_29_76030/76220_z3	0.2808948	0.491807
IFA	PON1_253_5402/5421	25.6158152	30.4351972
IFA	PON1_253_4301	21.5265258	29.3755552
IFA	PON1_253_54020_z3	12.2262258	18.6821024
IFA	PON1_253_43010_z3	184.797944	227.7171975
IFA	PON3_29_75310_z3	3.0430789	5.8493337
IFA	PON3_29_76210_z3	4.6268155	10.2021441
IFA	PON1_324_65030_z3	0.4699565	0.7920262
IFA	KNG1_169_5402	0.0027837	0.0033689
IFA	PON1_324_6502	49.4403839	63.8274665
IFA	PON1_324_5420	0.8756779	2.0658797
IFA	PON1_324_6501	3.9249468	6.3021571
IFA	SAA_94_55100_z3	0.0016891	0.0024032
IFA	SAA_94_54010_z3	0.007496	0.0089788
IFA	SAA_94_44010_z3	0.000459	0.0007009
IFA	SAA_94_53010_z3	0.0004467	0.0004924
IFA	SAA_94_43010_z3	0.0019437	0.0037609
IFA	SAA_94_54020_z3	0.0024335	0.004961
SQ-LNS	APOB_3224_54020_z3	0.0360742	0.0205106
SQ-LNS	APOB_3411_5401	0.0133048	0.031548
SQ-LNS	AACT_271_6512	0.0092851	0.0111823
SQ-LNS	AACT_271_7603	0.0433408	0.0328622
SQ-LNS	AACT_271_7602	0.0565462	0.0307353
SQ-LNS	AACT_271_6502	0.0037181	0.0036589

SQ-LNS	AACT_106_7603	0.0030077	0.0025984
SQ-LNS	APOB_185_52000_z3	0.5967682	0.886622
SQ-LNS	APOB_983_5401	0.0238097	0.013511
SQ-LNS	AACT_106_7604	0.0006532	0.0010299
SQ-LNS	A1BG_179_5421/5402	0.0187236	0.0051327
SQ-LNS	APOB_983_54020_z4	0.042181	0.0188127
SQ-LNS	AACT_127_5401	0.0019927	0.0020864
SQ-LNS	AACT_127_8500	0.0503658	0.0478195
SQ-LNS	A1AT_271_5412	0.0005837	0.0004937
SQ-LNS	A1AT_271_5402	0.066594	0.0372713
SQ-LNS	A1AT_271_55110_z3	0.0004226	0.000366
SQ-LNS	A1AT_271_5401	0.0006625	0.0005339
SQ-LNS	A1AT_271_6503	0.000254	0.0002328
SQ-LNS	APOA2_35_11020_z3	0.0014228	0.0012092
SQ-LNS	APOA2_35_11010_z2	0.0000171	0.0000361
SQ-LNS	A1AT_271MC_5412	0.0045508	0.0033261
SQ-LNS	A1AT_107_5401	0.0199933	0.013445
SQ-LNS	A1AT_107_5411	0.0384509	0.0131449
SQ-LNS	A1AT_271MC_5402	0.1519575	0.0496907
SQ-LNS	A1AT_107_6512	0.011594	0.0086922
SQ-LNS	A1AT_107_6502/6521	0.031491	0.0203581
SQ-LNS	A1AT_107_5402	0.0766724	0.0333366
SQ-LNS	A1AT_107_5412	0.0060377	0.0027078
SQ-LNS	A1AT_107_6513	0.010431	0.0068266
SQ-LNS	A1AT_107_6503	0.0316656	0.0147775
SQ-LNS	A1AT_107_6501/6520	0.0030173	0.0066414
SQ-LNS	APOA2_95_11010_z2	0.00039	0.0002525
SQ-LNS	APOA2_88_11010_z2	0.0003228	0.0002112
SQ-LNS	A1AT_70_5412	0.0008328	0.0004879
SQ-LNS	A1AT_70_5402	0.0708743	0.0403989
SQ-LNS	APOD_65_65210_z3	0.0000199	0.0000288
SQ-LNS	APOD_65_65030_z3	0.0000452	0.0000455
SQ-LNS	APOD_98_54100_z3	0.0011974	0.0018509
SQ-LNS	APOD_98_54000_z3	0.0001162	0.0000727
SQ-LNS	APOD_65_65020_z3	0.0000194	0.0000217
SQ-LNS	APOD_65_65010_z3	0.0000266	0.0000301
SQ-LNS	APOD_65_65020_z3_deamidated	0.0002	0.0002207
SQ-LNS	APOD_98_65010_z3	0.0000155	0.0000134
SQ-LNS	APOD_98_65110_z3	0.0000361	0.0000263
SQ-LNS	APOD_65_54010_z3	0.0000462	0.0000327
SQ-LNS	APOD_98_54110_z3	0.0002055	0.0001553
SQ-LNS	APOD_98_54020_z3	0.0000174	0.0000198
SQ-LNS	APOD_98_54010_z3	0.0000901	0.0000841

SQ-LNS	APOD_65_54020_z3	0.000037	0.000028
SQ-LNS	APOD_98_65130_z3	0.0000195	0.000026
SQ-LNS	APOD_98_54120_z3	0.0000516	0.0000964
SQ-LNS	APOD_65_76020_z3	0.0000268	0.0000183
SQ-LNS	APOD_98_5402	0.0005348	0.0005597
SQ-LNS	APOD_98_65320_z3	0.0000097	0.0000116
SQ-LNS	APOD_65_54200_z3	0.000054	0.0000444
SQ-LNS	APOD_65_76210_z3	0.0000289	0.0000282
SQ-LNS	APOD_65_65030_z3_deamidated	0.0000229	0.0000234
SQ-LNS	APOD_98_6520	0.0001724	0.0001188
SQ-LNS	APOD_98_6510	0.0003984	0.0003359
SQ-LNS	APOD_98_9800	0.0004614	0.0012463
SQ-LNS	APOD_98_6530	0.0005245	0.0005251
SQ-LNS	APOD_98_7600	0.0002207	0.0002143
SQ-LNS	APOC3_94AMC_12020_z3	0.0179115	0.038897
SQ-LNS	APOC3_94AMC_22100_z3	0.0007987	0.0010032
SQ-LNS	APOC3_94_1210	0.00149	0.0039736
SQ-LNS	APOC3_94_0310	0.0009661	0.0016544
SQ-LNS	APOC3_94_0300	0.0925521	0.0234368
SQ-LNS	APOC3_94AMC_11000_z3	0.0043751	0.0017712
SQ-LNS	APOC3_94MC_11010_z3	0.0230502	0.0090266
SQ-LNS	APOC3_94A_11010_z2	0.0009175	0.0004671
SQ-LNS	APOC3_94MC_1102	0.1548663	0.0333214
SQ-LNS	APOC3_94MC_11020_z3	0.0102879	0.0059773
SQ-LNS	APOC3_94_1101	0.1279492	0.0439271
SQ-LNS	APOC3_94A_11000_z2	0.0006756	0.0006089
SQ-LNS	APOC3_94Aoff_1101	0.0077636	0.0036551
SQ-LNS	APOC3_94_2110	0.0006797	0.0013118
SQ-LNS	APOC3_94Aoff_1102	0.0058798	0.0057642
SQ-LNS	APOC3_94_1102	0.0984933	0.0272781
SQ-LNS	APOC3_94A_11020_z2	0.0002145	0.000195
SQ-LNS	APOC3_94_1111	0.0023424	0.0042113
SQ-LNS	APOC3_94_1300	0.001765	0.0026773
SQ-LNS	APOC3_94_1202	0.0051276	0.011014
SQ-LNS	CLUS_291_5400	0.035016	0.0279911
SQ-LNS	CLUS_291_6503	0.0125713	0.0202466
SQ-LNS	CLUS_291_54020_z3	0.1369051	0.0434454
SQ-LNS	CLUS_291_5421/5402	0.104943	0.0327815
SQ-LNS	CLUS_291_5420/5401	0.0346106	0.0154522
SQ-LNS	CLUS_86_6503	0.001432	0.0012173
SQ-LNS	APOF_269_11010_z2	0.0911866	0.0614788
SQ-LNS	APOF_273/274_11010_z3	2.2598937	0.5417472
SQ-LNS	APOF_273/274_11020_z3	0.9932302	0.2866299

SQ-LNS	APOE_307/308_11010_z3	0.0397624	0.0098877
SQ-LNS	APOE_307/308_11010_z2	0.0011287	0.0003328
SQ-LNS	CLUS_374_6520/6501	0.104148	0.0551642
SQ-LNS	CLUS_374_5402/5421	0.10461	0.0369595
SQ-LNS	APOE_215_11010_z2	0.0050296	0.0048735
SQ-LNS	CLUS_374_5420/5401	1.3892337	0.6961809
SQ-LNS	APOE_215_11020_z2	0.0016964	0.0019215
SQ-LNS	CLUS_374_6512	0.108697	0.0527778
SQ-LNS	CO3_85_6200	0.4928755	0.3060726
SQ-LNS	CO3_85_5200	0.4637935	0.241497
SQ-LNS	CO3_85_7200	0.1941483	0.1536028
SQ-LNS	APOM_135_43010_z3	0.0006842	0.0002434
SQ-LNS	APOM_135_54010_z3	0.0004338	0.0001095
SQ-LNS	APOM_135_53010_z3	0.0001299	0.0000675
SQ-LNS	CO3_85_6301	1.2139166	1.3940368
SQ-LNS	APOM_135_54020_z3	0.0001889	0.0000913
SQ-LNS	APOM_135_44010_z3	0.0000361	0.0000243
SQ-LNS	APOM_135_55110_z3	0.0000398	0.0000249
SQ-LNS	C1S_174_5402	0.0105521	0.0069264
SQ-LNS	C1S_174_5401	0.0164874	0.0365968
SQ-LNS	APOM_135_5421	0.0001364	0.00015
SQ-LNS	APOM_135_8500	0.0000353	0.0000281
SQ-LNS	HEMO_240/246_5402	0.0016205	0.0017465
SQ-LNS	HEMO_187_6503	0.0192163	0.008273
SQ-LNS	HEMO_187_5402/5421	0.0917258	0.0185436
SQ-LNS	HEMO_187_5412/5431	0.0041695	0.0025208
SQ-LNS	FETUA_346_1101	0.6826585	0.1214871
SQ-LNS	FETUA_346_2200	0.0032741	0.0022572
SQ-LNS	FETUA_156_6411	0.0030369	0.0025073
SQ-LNS	FETUA_156_5400	0.0080833	0.0060188
SQ-LNS	FETUA_156_5401	0.0379221	0.0163555
SQ-LNS	HEP2_49_5402	0.3712908	0.2009065
SQ-LNS	FETUA_156_6410	0.0223969	0.0145957
SQ-LNS	FETUA_156_6502	0.0285029	0.0151165
SQ-LNS	FETUA_156_5402/5421	0.4969865	0.1013284
SQ-LNS	FETUA_156_6503	0.0296815	0.0133201
SQ-LNS	FETUA_176_6501	0.0427938	0.0269402
SQ-LNS	HEP2_49_5412	0.0324887	0.0298518
SQ-LNS	FETUA_156_6513	0.005065	0.0039551
SQ-LNS	HEMO_453_5420/5401	0.139898	0.0651389
SQ-LNS	FETUA_176_5401	0.0796742	0.0377109
SQ-LNS	FETUA_176_6512	0.0063726	0.0044113
SQ-LNS	FETUA_176_6502	0.0115723	0.0068503

SQ-LNS	FETUA_176_6513	0.0048559	0.0024456
SQ-LNS	FETUA_176_7600	0.0401939	0.0122964
SQ-LNS	FETUA_176_5412/5431	0.0208814	0.0069375
SQ-LNS	FETUA_176_5402/5421	0.1181136	0.0198114
SQ-LNS	FETUA_176_6503	0.0084927	0.0030904
SQ-LNS	HEMO_453_5402/5421	0.1609827	0.0477325
SQ-LNS	LCAT_108_65030_z3	0.0154938	0.0044167
SQ-LNS	KNG1_205_6503	0.0085932	0.0062304
SQ-LNS	KNG1_205_5402/5421	0.0049045	0.003644
SQ-LNS	KNG1_205_6512	0.007613	0.0088827
SQ-LNS	PON3_29_76030/76220_z3	0.1891634	0.2198464
SQ-LNS	PON1_253_5402/5421	22.9291195	22.2153279
SQ-LNS	PON1_253_4301	17.7064821	21.8488609
SQ-LNS	PON1_253_54020_z3	9.8524447	10.2595725
SQ-LNS	PON1_253_43010_z3	180.4528761	223.2800303
SQ-LNS	PON3_29_75310_z3	2.0986005	2.6136493
SQ-LNS	PON3_29_76210_z3	2.9688579	4.9107168
SQ-LNS	PON1_324_65030_z3	0.4423539	0.5318954
SQ-LNS	KNG1_169_5402	0.004139	0.006088
SQ-LNS	PON1_324_6502	44.6511212	61.8072691
SQ-LNS	PON1_324_5420	0.718557	1.1075797
SQ-LNS	PON1_324_6501	3.1246833	4.6988605
SQ-LNS	SAA_94_55100_z3	0.0014148	0.0013801
SQ-LNS	SAA_94_54010_z3	0.0061682	0.0056536
SQ-LNS	SAA_94_44010_z3	0.0003726	0.0004328
SQ-LNS	SAA_94_53010_z3	0.0005309	0.0005368
SQ-LNS	SAA_94_43010_z3	0.0012074	0.0012706
SQ-LNS	SAA_94_54020_z3	0.0020447	0.0020592

**Table S4.** Enrichment Analysis for glycopeptides in 21 HDL-associated proteins.						
**Protein**	**N**	**m**	**Number Glycopeptide Increased in SQ-LNS**	**Number Glycopeptide Decreased in SQ-LNS**	**P value Increased**	**P value Decreased**
A1AT	163	18	17	1	**0.0001244**	1
FETUA	163	19	16	3	**0.00393**	0.9994
AACT	163	8	8	0	**0.006192**	1
KNG1	163	4	4	0	0.08228	1
APOF	163	3	3	0	0.1549	1
APOM	163	8	6	2	0.1969	0.9479
C1S	163	2	2	0	0.2899	1
APOA2	163	4	3	1	0.3727	0.9177
A1BG	163	1	1	0	0.5399	1
LCAT	163	1	1	0	0.5399	1
HEMO	163	6	3	3	0.7315	0.5807
APOE	163	4	2	2	0.7475	0.6273
HEP2	163	2	1	1	0.7898	0.7101
APOB	163	5	2	3	0.8622	0.4246
APOD	163	27	12	15	0.9032	0.1899
CO3	163	4	1	3	0.9571	0.2525
SAA	163	6	1	5	0.9915	0.07248
CLUS	163	10	1	9	0.9997	**0.004316**
APOC3	163	20	4	16	0.9998	**0.001103**
PON3	163	3	0	3	1	0.0953
PON1	163	8	0	8	1	**0.001625**

The enrichment analysis is done using the hypergeometric distribution. **N** is the total number of glycopeptides; **m** is the total number of glycopeptides of a particular protein; **Number Glycopeptide Increase in SQ-LNS** is the number of glycopeptides that are increased in the SQ-LNS group; **Number Glycopeptide Decrease in SQ-LNS** is the total number of glycopeptides that are decreased in SQ-LNS group (higher in the IFA group); **P value Increased** can be interpreted as the probability that having x number of glycopeptides increased in the SQ-LNS group is by chance; **P value Decreased** can be interpreted as the probability that having x number of glycopeptides decreased in the SQ-LNS group is by chance. SQ-LNS, small-quantity lipid-based nutrient supplements; IFA, iron and folic acid.

Table S5. HDL composition and function and growth association analysis.

Predictor	Response	Model	95% CI	Pval
**Cholesterol Efflux Capacity**	HCZ18	Unadjusted	(-4.89e-02, 6.90e-02)	0.7358
		Adjusted	(-4.53e-02, 6.76e-02)	0.6957
	HCZ1812	Unadjusted	(-3.10e-02, 2.85e-02)	0.9345
		Adjusted	(-3.40e-02, 2.39e-02)	0.7289
	LAZ18	Unadjusted	(-8.91e-02, 3.30e-02)	0.3626
		Adjusted	(-8.84e-02, 3.47e-02)	0.3869
	LAZ1812	Unadjusted	(-1.22e-02, 3.95e-02)	0.2949
		Adjusted	(-2.10e-02, 2.62e-02)	0.8248
	WAZ18	Unadjusted	(-9.71e-02, 2.92e-02)	0.2884
		Adjusted	(-9.71e-02, 2.92e-02)	0.2884
	WAZ1812	Unadjusted	(-2.29e-02, 3.60e-02)	0.6567
		Adjusted	(-2.90e-02, 1.96e-02)	0.6988
	WLZ18	Unadjusted	(-9.10e-02, 3.60e-02)	0.3916
		Adjusted	(-6.90e-02, 6.35e-02)	0.9350
	WLZ1812	Unadjusted	(-3.46e-02, 3.71e-02)	0.9460
		Adjusted	(-3.64e-02, 2.17e-02)	0.6158
**Surface/core lipid ratio**	HCZ18	Unadjusted	(-4.72e-01, 7.25e-01)	0.6755
		Adjusted	(-4.85e-01, 6.72e-01)	0.7487
	HCZ1812	Unadjusted	(-3.67e-01, 2.47e-01)	0.6973
		Adjusted	(-3.46e-01, 2.60e-01)	0.7774
	LAZ18	Unadjusted	(-1.06e+00, 1.77e-01)	0.1598
		Adjusted	(-1.03e+00, 2.10e-01)	0.1921
	LAZ1812	Unadjusted	(-3.57e-01, 1.80e-01)	0.5124
		Adjusted	(-3.43e-01, 1.34e-01)	0.3845
	WAZ18	Unadjusted	(-1.04e+00, 2.43e-01)	0.2194
		Adjusted	(-1.04e+00, 2.43e-01)	0.2194
	WAZ1812	Unadjusted	(-5.06e-01, 9.57e-02)	0.1782
		Adjusted	(-4.37e-01, 5.36e-02)	0.1235
	WLZ18	Unadjusted	(-9.19e-01, 3.75e-01)	0.4054
		Adjusted	(-9.92e-01, 3.08e-01)	0.2975
	WLZ1812	Unadjusted	(-5.99e-01, 1.34e-01)	0.2094
		Adjusted	(-5.22e-01, 6.93e-02)	0.1311
**EOD_{18}**	HCZ18	Unadjusted	(-1.86e+00, 2.56e+00)	0.7524
		Adjusted	(-2.02e+00, 2.21e+00)	0.9299
	HCZ1812	Unadjusted	(-9.23e-02, 2.16e+00)	0.0714
		Adjusted	(-3.90e-01, 1.91e+00)	0.1913
	LAZ18	Unadjusted	(-3.01e+00, 1.64e+00)	0.5600
		Adjusted	(-2.71e+00, 2.03e+00)	0.7776
	LAZ1812	Unadjusted	(-2.44e-02, 1.97e+00)	0.0557
		Adjusted	(3.86e-02, 1.83e+00)	0.0412
	WAZ18	Unadjusted	(-2.60e+00, 2.23e+00)	0.8784

		Adjusted	(-2.60e+00, 2.23e+00)	0.8784
	WAZ1812	Unadjusted	(-8.38e-01, 1.48e+00)	0.5837
		Adjusted	(-1.00e+00, 9.05e-01)	0.9189
	WLZ18	Unadjusted	(-2.26e+00, 2.58e+00)	0.8940
		Adjusted	(-1.46e+00, 3.60e+00)	0.4026
	WLZ1812	Unadjusted	(-1.57e+00, 1.25e+00)	0.8232
		Adjusted	(-1.59e+00, 7.12e-01)	0.4495
**ACL**	HCZ18	Unadjusted	(-4.74e-01, 4.64e-01)	0.9825
		Adjusted	(-3.28e-01, 5.82e-01)	0.5802
	HCZ1812	Unadjusted	(-3.42e-01, 1.53e-01)	0.4496
		Adjusted	(-3.33e-01, 1.70e-01)	0.5202
	LAZ18	Unadjusted	(-1.45e-01, 8.34e-01)	0.1651
		Adjusted	(-1.14e-01, 8.75e-01)	0.1293
	LAZ1812	Unadjusted	(-3.55e-01, 8.38e-02)	0.2215
		Adjusted	(-2.25e-01, 1.83e-01)	0.8389
	WAZ18	Unadjusted	(-3.83e-01, 6.43e-01)	0.6160
		Adjusted	(-3.83e-01, 6.43e-01)	0.6160
	WAZ1812	Unadjusted	(-4.19e-01, 7.77e-02)	0.1751
		Adjusted	(-2.60e-01, 1.55e-01)	0.6161
	WLZ18	Unadjusted	(-5.59e-01, 4.71e-01)	0.8657
		Adjusted	(-6.72e-01, 3.58e-01)	0.5457
	WLZ1812	Unadjusted	(-4.48e-01, 1.59e-01)	0.3454
		Adjusted	(-3.33e-01, 1.61e-01)	0.4886
**APOA1**	HCZ18	Unadjusted	(-6.36e-08, 8.03e-07)	0.0934
		Adjusted	(-1.49e-07, 6.95e-07)	0.2017
	HCZ1812	Unadjusted	(-6.33e-08, 3.94e-07)	0.1538
		Adjusted	(-3.13e-08, 3.99e-07)	0.0925
	LAZ18	Unadjusted	(-2.29e-07, 6.79e-07)	0.3267
		Adjusted	(-1.23e-07, 7.60e-07)	0.1544
	LAZ1812	Unadjusted	(-1.43e-07, 2.59e-07)	0.5680
		Adjusted	(-7.87e-08, 2.80e-07)	0.2664
	WAZ18	Unadjusted	(-2.99e-07, 6.45e-07)	0.4685
		Adjusted	(-2.99e-07, 6.45e-07)	0.4685
	WAZ1812	Unadjusted	(5.04e-08, 4.89e-07)	0.0166
		Adjusted	(-2.47e-09, 3.80e-07)	0.0529
	WLZ18	Unadjusted	(-3.85e-07, 5.65e-07)	0.7067
		Adjusted	(-2.37e-07, 7.16e-07)	0.3199
	WLZ1812	Unadjusted	(8.43e-08, 6.14e-07)	0.0105
		Adjusted	(-2.08e-08, 4.46e-07)	0.0735
**SAA1**	HCZ18	Unadjusted	(-4.10e-05, 3.68e-05)	0.9161
		Adjusted	(-5.13e-05, 2.42e-05)	0.4770
	HCZ1812	Unadjusted	(-5.36e-06, 3.53e-05)	0.1462
		Adjusted	(-7.29e-06, 3.30e-05)	0.2070
	LAZ18	Unadjusted	(-5.52e-05, 2.43e-05)	0.4404

		Adjusted	(-4.99e-05, 3.14e-05)	0.6524
	LAZ1812	Unadjusted	(-1.25e-05, 2.30e-05)	0.5547
		Adjusted	(-1.74e-05, 1.48e-05)	0.8703
	WAZ18	Unadjusted	(-5.74e-05, 2.49e-05)	0.4342
		Adjusted	(-5.74e-05, 2.49e-05)	0.4342
	WAZ1812	Unadjusted	(1.70e-05, 5.36e-05)	0.0003
		Adjusted	(5.13e-06, 3.88e-05)	0.0114
	WLZ18	Unadjusted	(-5.30e-05, 2.97e-05)	0.5770
		Adjusted	(-4.36e-05, 3.94e-05)	0.9208
	WLZ1812	Unadjusted	(2.53e-05, 6.88e-05)	0.0001
		Adjusted	(1.26e-05, 5.17e-05)	0.0017
**SAA2**	HCZ18	Unadjusted	(-2.49e-05, 2.27e-05)	0.9242
		Adjusted	(-3.31e-05, 1.35e-05)	0.4060
	HCZ1812	Unadjusted	(-1.90e-06, 2.30e-05)	0.0953
		Adjusted	(-2.40e-06, 2.23e-05)	0.1123
	LAZ18	Unadjusted	(-3.24e-05, 1.60e-05)	0.5012
		Adjusted	(-3.13e-05, 1.76e-05)	0.5774
	LAZ1812	Unadjusted	(-8.29e-06, 1.34e-05)	0.6386
		Adjusted	(-9.72e-06, 9.74e-06)	0.9983
	WAZ18	Unadjusted	(-3.68e-05, 1.32e-05)	0.3507
		Adjusted	(-3.68e-05, 1.32e-05)	0.3507
	WAZ1812	Unadjusted	(1.16e-05, 3.37e-05)	0.0001
		Adjusted	(4.44e-06, 2.45e-05)	0.0054
	WLZ18	Unadjusted	(-3.56e-05, 1.47e-05)	0.4094
		Adjusted	(-2.91e-05, 2.17e-05)	0.7722
	WLZ1812	Unadjusted	(1.77e-05, 4.38e-05)	0.0000
		Adjusted	(8.88e-06, 3.27e-05)	0.0009
**APOL1**	HCZ18	Unadjusted	(-3.19e-05, 2.56e-05)	0.8278
		Adjusted	(-3.56e-05, 1.96e-05)	0.5676
	HCZ1812	Unadjusted	(1.12e-06, 2.98e-05)	0.0350
		Adjusted	(-2.17e-06, 2.61e-05)	0.0957
	LAZ18	Unadjusted	(-4.23e-05, 1.81e-05)	0.4278
		Adjusted	(-3.74e-05, 2.27e-05)	0.6274
	LAZ1812	Unadjusted	(-8.61e-06, 1.77e-05)	0.4933
		Adjusted	(-1.23e-05, 1.16e-05)	0.9510
	WAZ18	Unadjusted	(-3.09e-05, 3.19e-05)	0.9754
		Adjusted	(-3.09e-05, 3.19e-05)	0.9754
	WAZ1812	Unadjusted	(1.21e-05, 3.93e-05)	0.0004
		Adjusted	(6.56e-06, 3.02e-05)	0.0028
	WLZ18	Unadjusted	(-2.24e-05, 4.05e-05)	0.5678
		Adjusted	(-2.00e-05, 4.26e-05)	0.4738
	WLZ1812	Unadjusted	(1.65e-05, 4.92e-05)	0.0002
		Adjusted	(1.23e-05, 3.97e-05)	0.0003

ACL, average chain length; APOA1, apolipoprotein A-I; APOL1, apolipoprotein L1; CEC, cholesterol efflux capacity; EOD18, equivalent of double bond per 18 carbon; SAA1, serum amyloid A-1; SAA2, serum amyloid A-2; HCZ, head circumference for age z-score; LAZ, length for age z-score; WAZ, weight for age z-score; WLZ, weight for length z-score; 18, at 18 mo; 1812, changes in growth from 12 to 18 mo.

**Table S6**. Composition of supplements		
Nutrient	IFA	SQ-LNS
Ration, g/d		20
Total energy, kcal		118
Protein, g		2.6
Fat, g		10
Linoleic acid, g		4.59
α-Linolenic acid, g		0.59
Vitamin A, μg RE		800
Vitamin C, mg		100
Vitamin B-1, mg		2.8
Vitamin B-2, mg		2.8
Niacin, mg		36
Folic acid, μg	400	400
Pantothenic acid, mg		7
Vitamin B-6, mg		3.8
Vitamin B-12, μg		5.2
Vitamin D, IU		400
Vitamin E, mg		20
Vitamin K, μg		45
Iron, mg	60	20
Zinc, mg		30
Copper, mg		4
Calcium, mg		280
Phosphorus, mg		190
Potassium, mg		200
Magnesium, mg		65
Selenium, μg		130
Iodine, μg		250
Manganese, mg		2.6

IFA, iron and folic acid; SQ-LNS, small-quantity lipid-based nutrient supplements

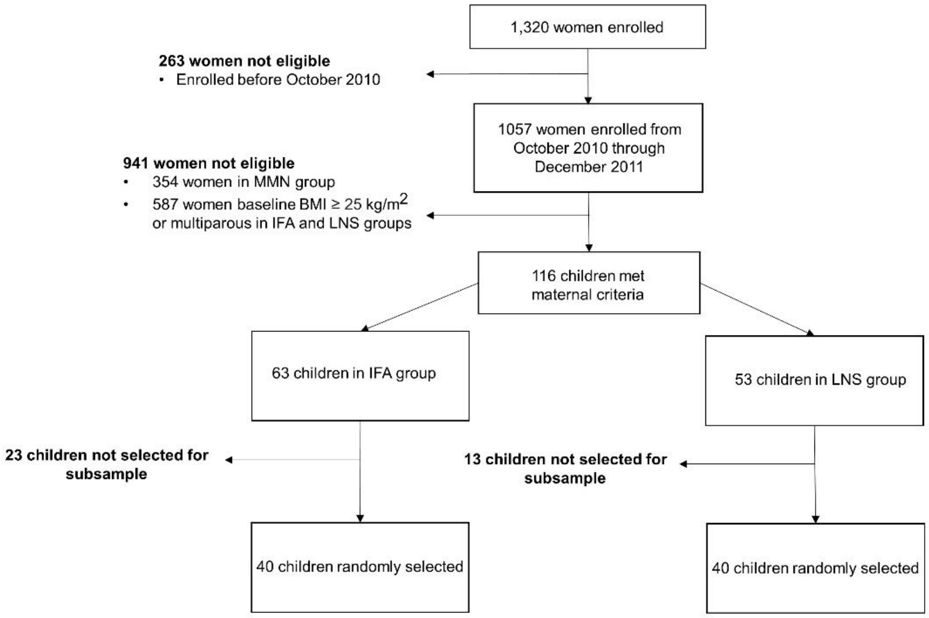

Supporting Information Figure S1. Flowchart of study profile

Supporting Information Method 1:

HDL Protein and Glycoprotein Identification

Isolated HDL fractions were diluted in a total of 100 µL with 50 mM ammonium bicarbonate buffer at pH 7.5. All reagents used for sample preparation were freshly prepared in a buffer of 50 mM ammonium bicarbonate. Proteins were then denatured with 2 µL of 550 mM dithiothreitol (Promega, Madison, WI) for 1 h at 65°C and alkylated with 4 µL of 450 mM iodoacetamide (Sigma-Aldrich, St. Louis, MO) for 30 min at room temperature away from light. Proteins were digested with 2 µg sequencing grade trypsin (Promega) for 18 h at 37°C. Samples were purified through Bond Elut C18 solid-phase extraction (Agilent, Santa Clara, CA), dried in a vacuum concentrator and reconstituted in 50 µL LC-MS grade water.

Samples were run on an Agilent 1290 Infinity II High-Performance Liquid Chromatography (HPLC) coupled to a Fusion Lumos MS/MS Orbitrap (Thermo Fisher Scientific). The HPLC was equipped with a 150 mm Agilent Zorbax Eclipse Plus C18 column with 1.8 µm particle size. Peptides and glycopeptides were eluted with a binary gradient of (A) 3% acetonitrile with 0.1% formic acid in water, and (B) 90% acetonitrile with 0.1% formic acid in water. The HPLC was set at a flow rate of 0.3 mL/min and programmed to ramp from 0% to 20% B in 20 min, 30% at 40 min, 44% at 47 min, and 100% at 48 min followed by a flushing and equilibration cycle. The electrospray ionization (ESI) voltage was set to 3500 V in the positive mode.

The Orbitrap was operated in positive mode with a precursor scan resolution of 60,000 and a range of 350-2000 m/z. Fragmentation was accomplished by stepped High-energy Collisional Dissociation (HCD). The collision energy was set at 30% and stepped at 10%. Ions for fragmentation were filtered to include a precursor mass range of 700-2000 m/z and charge states between 2-6.

Peptides and glycopeptides were identified with Byonic software (Protein Metrics Inc) from the Orbitrap MS data. For glycopeptide identification, a database of protein sequences and a library of glycan compositions are required as inputs. The human proteome database was

downloaded from Uniprot.org. We used in-house libraries for N-glycan and O-glycan compositions. (Glyco)peptides were identified based on the accurate mass of the precursor ions with tolerance set at 10 ppm and by matching MS/MS fragmentation with theoretical MS/MS spectra generated from in silico digestion of the provided protein database.

A total of 33 HDL-associated proteins were monitored in this study including apolipoprotein A-I (APOA1), apolipoprotein(a) (LPA), apolipoprotein A-II (APOA2), apolipoprotein A-IV (APOA4), apolipoprotein A-V (APOA5), apolipoprotein C-II (APOC2), apolipoprotein C-IV (APOC4), apolipoprotein F (APOF), apolipoprotein L1 (APOL1), haptoglobin-related protein (HPR), phosphatidylcholine-sterol acyltransferase (LCAT), phospholipid transfer protein (PLTP), alpha-1-antitrypsin (A1AT), alpha-1B-glycoprotein (A1BG), alpha-1-antichymotrypsin (AACT), apolipoprotein B-100 (APOB100), apolipoprotein C-I (APOC1), apolipoprotein C-III (APOC3), apolipoprotein D (APOD), apolipoprotein E (APOE), beta-2-glycoprotein 1 (APOH), apolipoprotein M (APOM), complement C1s subcomponent (C1S), clusterin (CLUS or APOJ), complement C3 (C3), alpha-2-HS-glycoprotein (FETUA or AHSG), hemopexin (HPX), heparin cofactor 2 (HCF2), kininogen-1 (KNG1), serum paraoxonase/arylesterase 1 (PON1), serum amyloid A-4 (SAA4) , serum amyloid A-1 (SAA1) , serum amyloid A-2 (SAA2).

Supporting Information Method 2:

Targeted glycoproteomics analysis

Tryptic digestion of the HDL samples was done in a 96-well format to facilitate batch processing. Samples were randomized before plating. All reagents were freshly prepared in a buffer of 50 mM ammonium bicarbonate. A sample volume of 10 μL purified HDL was used for tryptic digestion. After every 20 samples, 10 μL of commercially available human serum (Sigma-Aldrich) was also digested to serve as sample preparation controls. Protein standards (APOA1, APOC1, APOD, APOE, CLUS; all from Sigma-Aldrich) were mixed in known amounts (250, 250, 125, 200, and 125 μg/mL, respectively) and digested with the batch to serve as calibration standards. Serial dilution of the digested protein mixture provided the calibration curve for absolute quantitation of the 5 proteins. The digested mixture was diluted by factors of 160, 80, 40, 20, 16, 8, 4, 2, and 1 to obtain 9 calibration standards, from which calibration curves spanning 4 orders of magnitude were calculated.

After pipetting the samples, controls, and standards onto the 96-well plate, 10 μL of 100 mM dithiothreitol was added to each well to reduce the protein disulfide bonds. Protein denaturation was continued by heating in a water bath for 1 h at 65°C. Samples were then alkylated with 5 μL of 360 mM iodoacetamide for 30 min at room temperature away from light. Excess iodoacetamide was quenched with 5 μL of 100 mM dithiothreitol. Proteins were digested with 10 μl of 200 μg/mL sequencing grade trypsin for 18 h at 37°C. The digestion was stopped by acidifying the solution with 5 μL 10% (v/v) formic acid (Fluka). To account for batch variability and possible run-order effects, 5 μL of a 1 μg/mL synthetic peptide with sequence RPAIAINNPYVPR (Bionexus, Oakland, CA) was added as an internal standard. The final volume of the HDL digest is 50 μL, a 5-fold dilution of the purified HDL solution. The samples were injected into the LC-MS instrument without further cleanup.

(Glyco)peptides were quantified on an Agilent 1290 Infinity II LC system coupled to an Agilent 6495B Triple Quadrupole MS. Injection volumes were set at 5 μL for protein standards

and HDL samples, and 1 µL for serum digests. A pooled sample of the digested serum was run after every 10 HDL samples to serve as quality control (QC). They were used to monitor the stability of the instrument and the reproducibility of the batch analysis.

The HPLC was equipped with a 150 mm Agilent Zorbax Eclipse Plus C18 column with 1.8 µm particle size. A C18 column guard was used to protect the column from the buildup of lipids and other hydrophobic substances in the sample. A binary gradient of (A) 3% acetonitrile with 0.1% formic acid in water and (B) 90% acetonitrile with 0.1% formic acid in water was set at a flow rate of 0.5 mL/min. The HPLC pump parameters were programmed to ramp from 0% to 20% B in 20 min, 30% at 40 min, 44% at 47 min, and 100% at 48 min followed by 12 min column flushing cycle with 100% B and 7 min equilibration at 100% A. The Electrospray Ionization (ESI) voltage was set to 3500 V in the positive mode.

A transition list for target analytes was created by combining previously reported transitions (25,26) with new transitions selected from the Orbitrap analysis. The transition list included 47 peptides and 163 glycopeptides from 33 proteins. The instrument was run on Dynamic Multiple Reaction Monitoring (DMRM) mode to minimize the number of transitions being monitored at each scan cycle. For peptides, at least 2 product ions were selected for monitoring. Quantitation was based on the area of the more abundant product ion while the others are for qualitative identification. Product ions for glycopeptides were based on diagnostic glycan fragments. Glycans yield characteristic oxonium ions after Collision Induced Dissociation (CID) with mass-to-charge ratios (m/z) of 204.08, 274.09, and 366.14 for N-acetylhexosamine (HexNAc), N-acetylneuraminic acid (NeuAc) with loss of H2O, and hexose + HexNAc (Hex1HexNAc1) respectively.
